# TROP2 targeting reveals therapy-driven cell state dynamics in colorectal cancer

**DOI:** 10.1038/s41586-026-10705-2

**Published:** 2026-07-01

**Authors:** Nuria Vaquero-Siguero, Nikolaos Georgakopoulos, Maria C. Puschhof, Ioannis Chiotakakos, Jasmin Meier, Sigrid K. Fey, Gabriele Diamante, Manuel Mastel, Aitana Guiseris-Martinez, Guillaume Belthier, Nikolai Schleußner, Julia Volk, Carolin Artmann, Bryce Lim, Ronald Koschny, Cyrill Wehling, Kyanna S. Ouyang, Michael Günther, Solveig Kuss, Paula Hoffmeister, Moritz Mall, Jens Neumann, Steffen Ormanns, Martin Schneider, Thomas Schmidt, Jens Puschhof, Andreas Trumpp, Jacco van Rheenen, Julio Saez-Rodriguez, Bruno C. Köhler, Rene Jackstadt

**Affiliations:** 1 Heidelberg Institute for Stem Cell Technology and Experimental Medicine (HI-STEM gGmbH), Heidelberg, Germany; 2 Cancer Progression and Metastasis Group, German Cancer Research Center (DKFZ) and DKFZ–ZMBH Alliance, Heidelberg, Germany; 3 Faculty of Biosciences, Heidelberg University, Heidelberg, Germany; 4 Faculty of Medicine, Heidelberg University, Heidelberg, Germany; 5 Division of Stem Cells and Cancer, German Cancer Research Center, Heidelberg, Germany; 6 Institute for Computational Biomedicine, Heidelberg University Hospital, Heidelberg, Germany; 7 Division of Molecular Pathology, Oncode Institute, Netherlands Cancer Institute, Amsterdam, the Netherlands; 8 Department of General, Visceral and Transplant Surgery, University Hospital of Heidelberg, Heidelberg, Germany; 9 Division of Cell Fate Engineering and Disease Modeling, German Cancer Research Center (DKFZ) and DKFZ–ZMBH Alliance, Heidelberg, Germany; 10 Hector Institute for Translational Brain Research, Heidelberg, Germany; 11 Central Institute of Mental Health, Medical Faculty Mannheim, Heidelberg University, Mannheim, Germany; 12 Interdisciplinary Endoscopy Center (IEZ)/Department of Gastroenterology, University Hospital Heidelberg, Heidelberg, Germany; 13 Department of Gastroenterology, Section of Translational Endoscopic Research, University Hospital Heidelberg, Heidelberg, Germany; 14 Junior Research Group Epithelium Microenvironment Interactions (EMIL), German Cancer Research Center, Heidelberg, Germany; 15 DKFZ Hector Cancer Institute at the University Medical Center Mannheim, Mannheim, Germany; 16 Institute of General Pathology, Medical University Innsbruck, Innsbruck, Austria; 17 Institute of Pathology, Faculty of Medicine, LMU Munich, Munich, Germany; 18 Department of Medical Oncology, Heidelberg University Hospital, Medical Faculty Heidelberg, Heidelberg University, Heidelberg, Germany; 19 National Center for Tumor Diseases (NCT), NCT Heidelberg, German Cancer Research Center (DKFZ) and Heidelberg University Hospital, Heidelberg, Germany; 20 Section of Precision Oncology and Clinical Trials, Department of Hematology and Oncology, University Medical Center Schleswig-Holstein and University Cancer Center Schleswig-Holstein, Lübeck, Germany; 21 Institute of Pathology, Paracelsus Medical University, Nuremberg Hospital, Nuremberg, Germany; 22 Department of General, Visceral, Thoracic, and Transplant Surgery, University of Giessen, Giessen, Germany; 23 Department of General, Visceral, Thorax and Transplant Surgery, University Hospital of Cologne, Cologne, Germany; 24 German Cancer Consortium (DKTK), German Cancer Research Centre (DKFZ), Heidelberg, Germany; 25 European Molecular Biology Laboratory, European Bioinformatics Institute (EMBL-EBI), Hinxton, UK

**Keywords:** Colorectal cancer, Cancer stem cells, Intestinal stem cells

## Abstract

Metastasis remains the leading cause of cancer-related mortality and is driven by pronounced tumour cell plasticity^1^. Here we identify the transmembrane glycoprotein trophoblast cell-surface antigen 2 (TROP2) as a marker of poor-prognosis colorectal cancer (CRC) associated with WNT^low^, fetal-like tumour cell states that are linked to metastasis and therapy resistance. Functional analyses demonstrate that TROP2^+^ cells exhibit context-dependent stem-like capacity and the ability to initiate metastatic outgrowth. Given that these detrimental tumour states converge on the cell-surface antigen TROP2, we explored therapeutic targeting of this cell population using clinically relevant TROP2-directed antibody–drug conjugates. Time-resolved analyses reveal therapy-associated dynamics in tumour cell state composition between WNT^hi^ LGR5^+^ states and WNT^low^TROP2^+^ fetal-like states. Conventional chemotherapy promotes the induction of TROP2-expressing cells, whereas TROP2 antibody–drug conjugates selectively target these populations and remodel the tumour cell state landscape. Exploiting this plasticity, combined chemotherapy and TROP2 targeting enhances anti-tumour efficacy in patient-derived models. Together, our findings identify TROP2 as a therapeutic vulnerability of CRC and highlight the importance of targeting tumour cell states to improve therapeutic efficacy and overcome resistance in advanced disease.

## Abstract (web summary)

TROP2 marks poor-prognosis colorectal cancer cells with fetal-like, therapy-resistant states, and therapeutic targeting of TROP2 with antibody–drug conjugates in combination with chemotherapy enhances treatment efficacy by modulating tumour cell plasticity and overcoming resistance.

## Main

Colorectal cancer (CRC) is the second leading cause of cancer-associated deaths worldwide. Its high fatality rate is primarily attributable to the presence of disseminated tumour cells at the time of diagnosis and ineffective systemic therapies^2^. The standard therapeutic intervention for microsatellite-stable CRC, which accounts for around 90% of CRC cases, is a combination of cytostatic agents, such as 5-fluorouracil (5-FU) with either irinotecan (FOLFIRI) or oxaliplatin (FOLFOX). Nevertheless, cellular plasticity seems to be a critical driver of therapy resistance. Furthermore, targeting vascular endothelial growth factor receptor or epidermal growth factor receptor increases clinical efficacy^3,4^. Approval of these combinatorial treatments was granted more than a decade ago^3^; however, the 12% 5-year overall survival rate for stage IV patients underscores the unmet need for improved treatment options for patients with advanced CRC^5^.

Poor-prognosis CRC remains a drastic clinical challenge. Molecular characterization revealed that tumours with poor prognosis are particularly characterized by low WNT signalling and belong to the serrated subtype^6–8^. Further transcriptional subtyping approaches uncovered the existence of four consensus molecular subtypes (CMSs)^9^. Recently, single-cell sequencing refined the CMS into intrinsic CMS (iCMS), which highlighted that iCMS3 confers cancer cells with poor-prognosis features^10^. Another strategy to subdivide CRC was taken by pathway-derived subtypes (PDSs), in which PDS3 showed the worst prognosis in locally advanced disease^11^. All these poor-prognosis treatment-refractory subtypes display similarly low WNT-pathway activity. These classifications have not yet entered clinical space in terms of patient stratification or therapeutic approach.

Cancer stem cells (CSCs) are cancer cell populations characterized by enhanced self-renewal capacities critical for cancer maintenance, therapy resistance and metastasis^12^. Derived from adult stem cells in the intestine, *LGR5*-expressing cells were initially defined as the cell of origin and CSCs in CRC^13,14^. Depletion of LGR5^+^ cancer cells revealed cellular plasticity with fast replenishment of the LGR5 CSC pool in a context-dependent manner^14,15^, driven by mTOR signalling activation^16^. Depletion of LGR5^+^ cells in liver metastases led to significant tumour reduction^15^. Tracing of cellular dynamics in the metastatic cascade by intravital imaging revealed that tumour cells leaving the primary tumour are mostly devoid of *LGR5* expression^17^ while showing activation of YAP signalling^18^. Similarly, drug-tolerant persister cells regain expression of fetal-like cell markers^19,20^. Notably, fetal-like cancer cells are detected in many disease-relevant processes that are critical for patient outcomes, such as metastasis, whereas LGR5^+^ adult intestinal stem cells (ISCs) are absent^21^. Furthermore, those cells gain expression of *L1CAM* or *EMP1*, which also identify populations in a regenerative, MAPK-active or high-relapse cell (HRC) state^22–26^.

Recent years have seen rapidly growing interest in antibody–drug conjugates (ADCs) as a targeted therapeutic strategy across multiple cancer types^27,28^. Trophoblast cell-surface antigen 2 (TROP2) is a transmembrane glycoprotein originally identified in mouse trophoblasts and encoded by the tumour-associated calcium signal transducer 2 (*TACSTD2*) gene. Notably, TROP2 has been shown to be abundantly expressed in various carcinomas while remaining absent in most adult tissues^29^. The description of the TROP2 antigen has sparked high interest in the development of TROP2-directed ADCs. These ADCs, such as the first-in-class product sacituzumab govitecan (SG/Trodelvy) or sacituzumab tirumotecan (Sac-TMT), consist of a humanized anti-TROP2 monoclonal antibody (hRS7) conjugated with topoisomerase inhibitors SN-38 or KL610023 (refs. ^30,31^). SG has already been granted regular approval for patients with metastatic breast cancer in 2021 (ref. ^32^). The success of TROP2-targeting ADCs led to the launching of numerous TROP2–ADCs that are in clinical development, ranging from phases I to III in various cancers^21^.

The emergence of distinct cell states during CRC progression and therapy resistance highlights the need to therapeutically target these states. Our profiling of human CRC identified TROP2 as a clinically relevant candidate associated with CRC progression, fetal-like cells and the HRC state. Functional investigations have demonstrated that TROP2 marks tumour cells with enhanced self-renewal capacity in those devoid of WNT signalling. We observed a binary split between LGR5^+^ and TROP2^+^ cell states, with notable plasticity between them. Challenging CRCs with chemotherapy enhanced TROP2 expression. By contrast, combination treatment with TROP2-targeting ADCs and chemotherapy showed enhanced efficacy. Taken together, we demonstrate that targeting of specific cell states mediates therapy resistance, which can be overcome with mechanistically informed cell-state-selective combination therapies.

## TROP2 defines poor-prognosis CRC cells

**Fig. 1:**
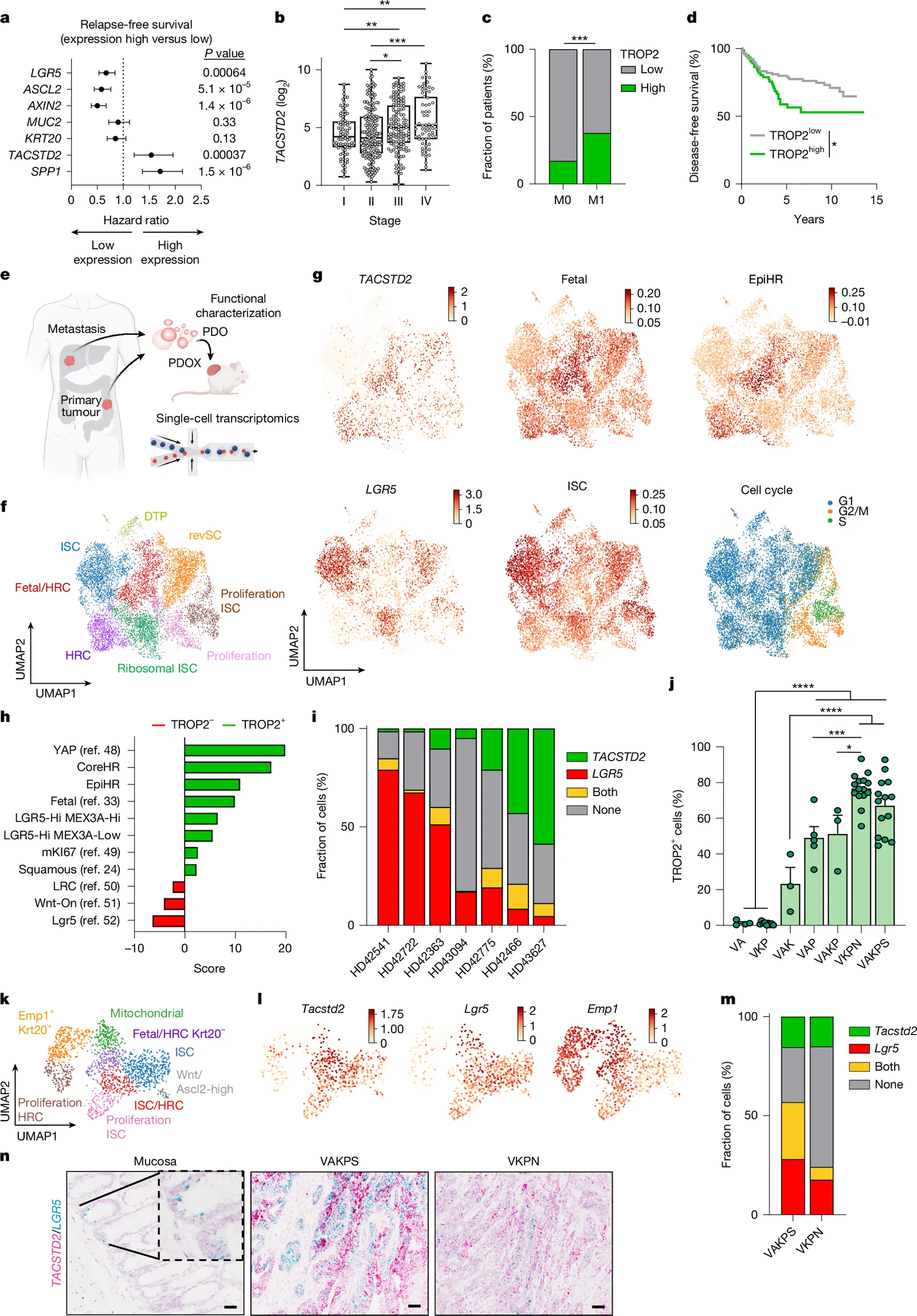
**TROP2 defines poor-prognosis CRC cells.** **a**, Forest plot showing relapse-free survival of indicated genes in 1,336 patients with CRC from 16 publicly available datasets. **b**, *TACSTD2* expression analysis across stages of human CRC from the The Cancer Genome Atlas Colon Adenocarcinoma (TCGA-COAD) dataset (*n* = 468). Pairwise comparisons were performed using the Wilcoxon rank-sum test. **c**, TROP2 expression in a case–control study of non-metastatic (M0) and metastatic (M1) CRC (*n* = 246). Statistical analysis was performed using Pearson’s *χ*^2^ test. **d**, Kaplan–Meier curve displaying disease-free survival of patients with CRC (UICC stage II, pT3/4) (*n* = 189). Statistical analysis was performed using the log-rank (Mantel–Cox) test. **e**, Schematic indicating the strategy of biospecimen generation and subsequent scRNA-seq. **f**, Uniform manifold approximation and projection (UMAP) of annotated cancer cell states (*n* = 10,135 cells; seven donors). **g**, UMAP representation of normalized gene expression levels of gene set scores. Colour scale capped at 99th percentile. **h**, Enrichment analysis results of public gene sets significantly changing between TROP2^+^ and TROP2^−^ cells from **f** using a univariate linear model. **i**, Tumour composition showing LGR5^+^ and TROP2^+^ abundance across donors from **f**. **j**, Flow cytometry analysis of TROP2 levels in MDOs (*n* = 4, 9, 3, 5, 3, 15 and 14). Statistical analysis was performed using ordinary one-way analysis of variance (ANOVA). **k**, UMAP embedding of annotated mouse epithelial tumour cells (*n* = 1,573 cells; three mice each for VAKPS and VKPN). **l**, UMAP representation with marker gene expression from **k**. Colour scale capped at 99th percentile. **m**, Tumour composition showing LGR5^+^ and TROP2^+^ abundance across GEMMs from **k**. **n**, In situ hybridization of normal or tumour tissue from GEMMs. Scale bar, 50 µm (**n**). *P < 0.05, **P < 0.01, ***P < 0.001, ****P < 0.0001. Schematic in **e** created in BioRender; Jackstadt, R. https://biorender.com/x995kgp (2026).

**Extended Data Fig. 1:**
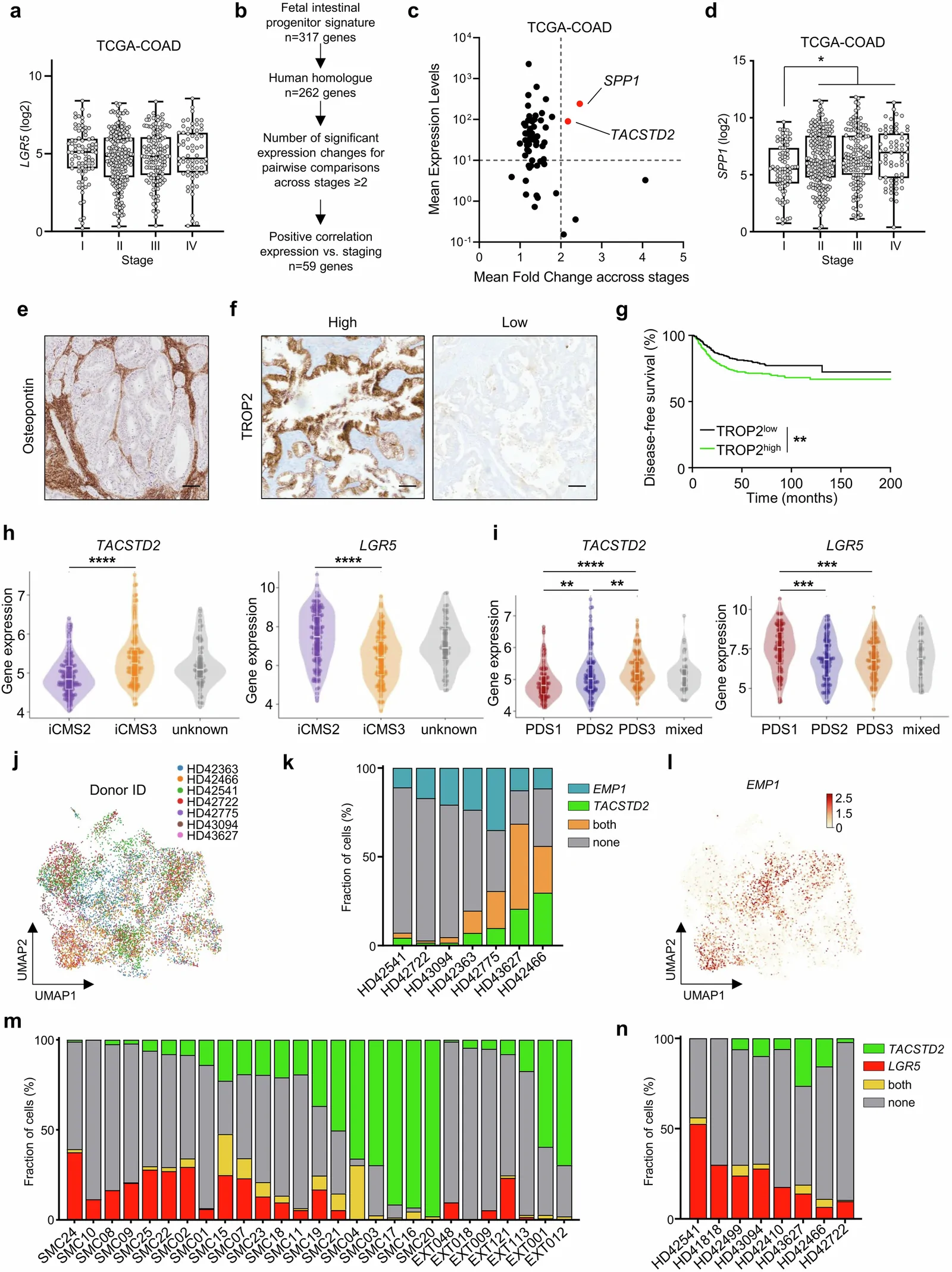
TROP2 defines poor prognosis human colorectal cancer. **a**, Expression levels of *LGR5* across stages in the TCGA colon adenocarcinoma (COAD) cohort (n = 468). There are no statistically significant changes as tested using a Wilcoxon rank sum test (pairwise comparisons, *p*-values > 0.05). **b**, Filtering strategy for human fetal marker genes associated with late-stage CRC. **c**, Gene expression level versus fold change in gene expression across clinical stages in the TCGA-COAD cohort for all fetal genes selected in **b**. **d**, Expression level of *SPP1* across stages of human CRC in the TCGA-COAD cohort (n = 468). Pairwise comparisons were done using a Wilcoxon rank sum test. **e**, Immunohistochemistry of osteopontin in human CRC. Scale bar 100 µm. **f**, Representative TROP2 immunohistochemistry images of human CRC of the different groups split by TROP2 expression levels depicted in Fig. 1d. Scale bar 100 µm. **g**, Kaplan-Meier plot of CRC patients from 16 publicly available datasets segregated according to TROP2 expression levels (n = 1,336). Statistical analysis was performed using the log-rank (Mantel–Cox) test. **h**, Violin plots indicating gene expression levels stratified by iCMS status (n = 360). Statistical analysis was performed using a two-sided Wilcoxon rank-sum test. **i**, Violin plots depicting gene expression levels for different PDS (n = 360). Statistical analysis was performed using a two-sided Wilcoxon rank-sum test. **j**, UMAP representation colour-coded by donor identity, related to Fig. 1f. **k**, Tumour composition with respect to *EMP1*^+^ and *TACSTD2*^+^ subpopulations split by donor identity from Fig. 1f. **l**, UMAP representation indicating expression levels of *EMP1* in tumour cells. Colour scale capped at 99.5th percentile. **m**, Tumour composition with respect to cell subpopulations in samples from^51^. **n**, Tumour composition with respect to cell subpopulations as measured in tissue samples from CRC patients, matching Fig. 1f.

Aggressive CRC has been associated with tumours characterized by low WNT-signalling activity^4,7,8^. To define highly metastatic and drug-resistant cells in CRC, we analysed the WNT-signalling targets and stem cell markers *LGR5*, *ASCL2* and *AXIN2* in publicly available datasets. Expression of these genes was significantly associated with prolonged relapse-free survival (Fig. 1a and Extended Data Fig. 1a). To identify genes associated with shorter relapse-free survival and CRC progression, we next applied a fetal gene signature, which was previously shown to be associated with therapy resistance and enhanced in WNT^low^ serrated CRC^4,7,8,33^. We identified two fetal genes highly expressed in CRC and positively correlated with advanced tumour stage: *TACSTD2*, which encodes TROP2, and *SPP1*, which encodes osteopontin (Fig. 1a,b and Extended Data Fig. 1b–d). We focused on the analysis of the epithelium-expressed transmembrane protein TROP2, as osteopontin is a protein abundantly expressed in the stromal compartment of CRC (Extended Data Fig. 1e). In a case–control cohort of 246 patients matched according to tumour stage, grading and primary tumour site, we found that TROP2 expression significantly correlated with distant metastasis compared with the control group (37.8% versus 17.1%) (Fig. 1c and Supplementary Table 1). Further, high TROP2 expression correlated with shorter disease-free survival in advanced localized CRCs (Union for International Cancer Control (UICC) stage II, pT3/4) and in 16 publicly available cohorts comprising 1,336 patients with CRC (Fig. 1d, Extended Data Fig. 1f,g and Supplementary Table 2). In line with this, *TACSTD2* expression was increased in the poor-prognosis subtypes iCMS3 and PDS3 (refs. ^10,11^), whereas *LGR5* was negatively correlated (Extended Data Fig. 1h,i). To further define TROP2 expression and cancer cell programs, we performed single-cell RNA sequencing (scRNA-seq) on a collection of CRC primary tumours and liver metastases. For functional characterization, we generated patient-derived organoids (PDOs) and PDO xenografts (PDOXs), which were analysed by scRNA-seq (Fig. 1e and Extended Data Fig. 1j). Cell cluster annotation of the epithelial tumour compartment revealed the presence of previously defined clusters, such as revival stem cells (marked by *CLU*), proliferative and non-proliferative *LGR5* ISC clusters and a distinct cluster enriched for *MEX3A*, which was associated with drug-tolerant persister (DTP) cells. Furthermore, two clusters with increased *TACSTD2* expression were identified: one enriched for epithelial HRCs^22^ and the other for fetal-like cells (fetal/HRC)^33^ (Fig. 1f and Supplementary Table 3). Although *TACSTD2* and *EMP1* are expressed in similar clusters, only a small fraction of cells were double-positive (Fig. 1g and Extended Data Fig. 1k,l). Notably, *TACSTD2*-expressing and *LGR5*-expressing cells seemed to define two distinct cell populations in our datasets and in public datasets (Fig. 1g–i and Extended Data Fig. 1m,n). Taken together, these data show that TROP2 defines a cell population associated with poor prognosis in human CRC.

**Extended Data Fig. 2:**
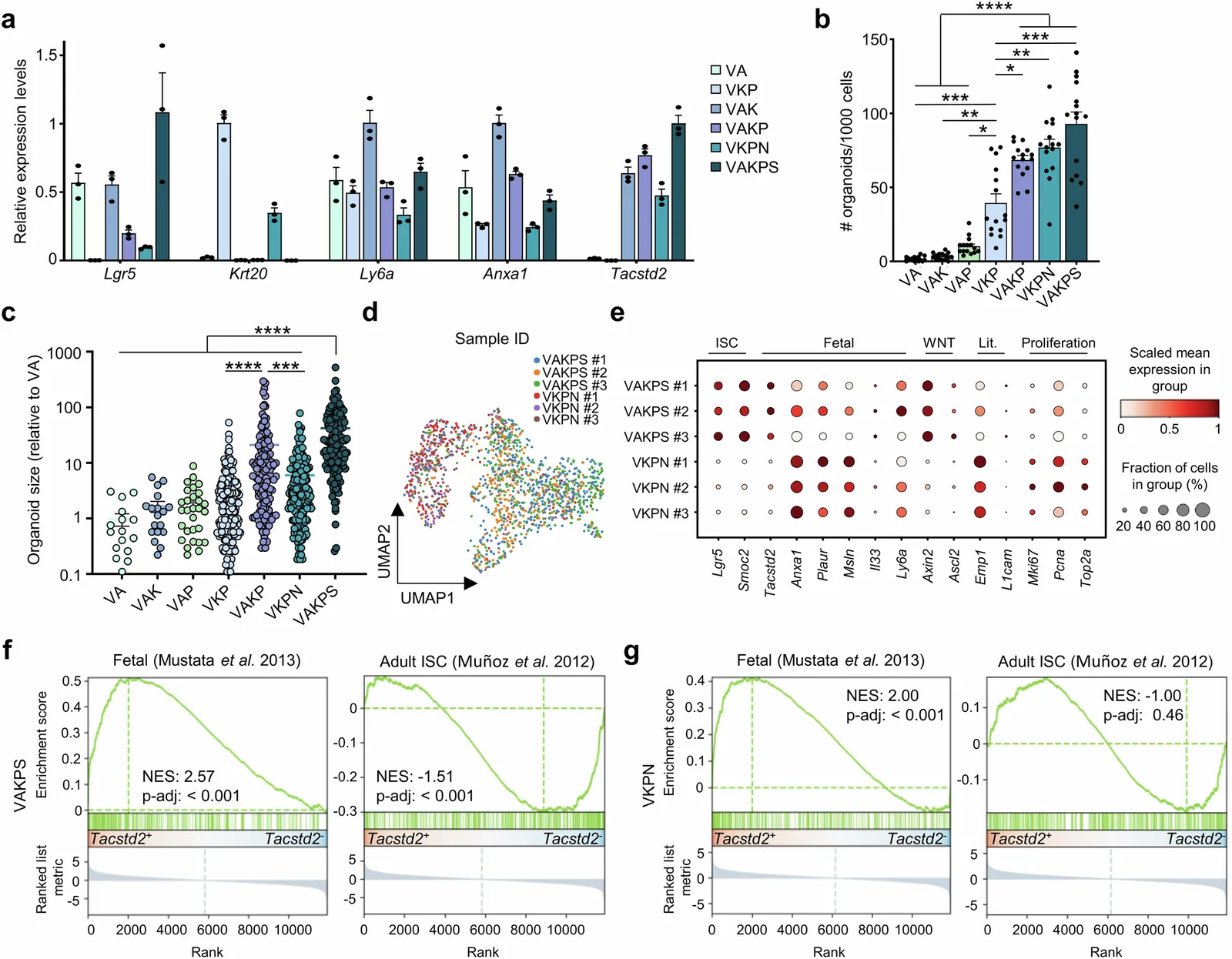
TROP2 in mouse models of colorectal cancer. **a**, Quantitative real-time PCR results of MDOs for indicated genes (n = 3). **b**, Clonogenicity assay of MDOs five days after seeding (n = 15 individual wells from three independent experiments). Statistical analysis was performed using ordinary one-way ANOVA. **c**, Size of the MDOs from **b**. Statistical analysis was performed using ordinary one-way ANOVA. **d**, UMAP showing the distribution of CRC mouse models and replicates, related to Fig. 1k (n = 1,573 cells). **e**, Dot plot showing scaled average gene expression across mouse tumours from **d**. Lit.= in literature described marker genes. **f**, and **g**, Gene set enrichment analysis (GSEA) of indicated gene sets on *Tacstd*2^+^ and *Tacstd2*^−^ pseudobulk cell populations of VAKPS **f** and VKPN **g** derived tumours.

We generated an array of genetically engineered mouse models (GEMMs) that define progression to CRC through either the classical route or the serrated route^7^. These models represent an excellent toolbox to investigate the stepwise progression towards advanced CRC. Consequently, we profiled the expression of TROP2 in genetically defined mouse-derived organoids (MDOs). In these MDOs ranging from benign models (*Villin1*–Cre^ER^ *Apc*^*fl/fl*^, *Villin1*–Cre^ER^ *Apc*^*fl/fl*^ *Trp53*^*fl/fl*^, *Villin1*–Cre^ER^ *Apc*^*fl/fl*^ *Kras*^*G12D/+*^) to invasive adenocarcinoma models (*Villin1*–Cre^ER^ *Apc*^*fl/+*^ *Kras*^*G12D/+*^ *Trp53*^*fl/fl*^ (VAKP), *Villin1*–Cre^ER^ *Kras*^*G12D/+*^ *Trp53*^*fl/fl*^ (VKP)) to highly metastatic adenocarcinoma models (*Villin1*–Cre^ER^ *Apc*^*fl/+*^ *Kras*^*G12D/+*^ *Trp53*^*fl/fl*^ *Smad4*^*fl/fl*^ (VAKPS), *Villin1*–Cre^ER^ *Kras*^*G12D/+*^ *Trp53*^*fl/fl*^ *Rosa26*^*N1icd/+*^ (VKPN)), we found higher TROP2 levels with increased oncogenic burden (Fig. 1j and Extended Data Fig. 2a). By contrast, *Lgr5* or fetal-like markers, such as *Ly6a* or *Anxa1*, did not follow this stepwise increase (Extended Data Fig. 2a). In line with increased TROP2 expression, we also detected increased seeding capacities of single cells and organoid sizes (Extended Data Fig. 2b,c). Single-cell transcriptomics of tumours from the metastatic models VKPN and VAKPS confirmed the existence of independent *Tacstd2*^*+*^, *Lgr5*^*+*^ or *Emp1*^*+*^ cells and the association of *Tacstd2* expression with fetal-like cells (Fig. 1k–n and Extended Data Fig. 2d–g). Together, we identified TROP2 as a marker of poor-prognosis cell states in human and mouse CRCs.

## TROP2 marks CSCs of WNT^low^ CRC

**Fig. 2:**
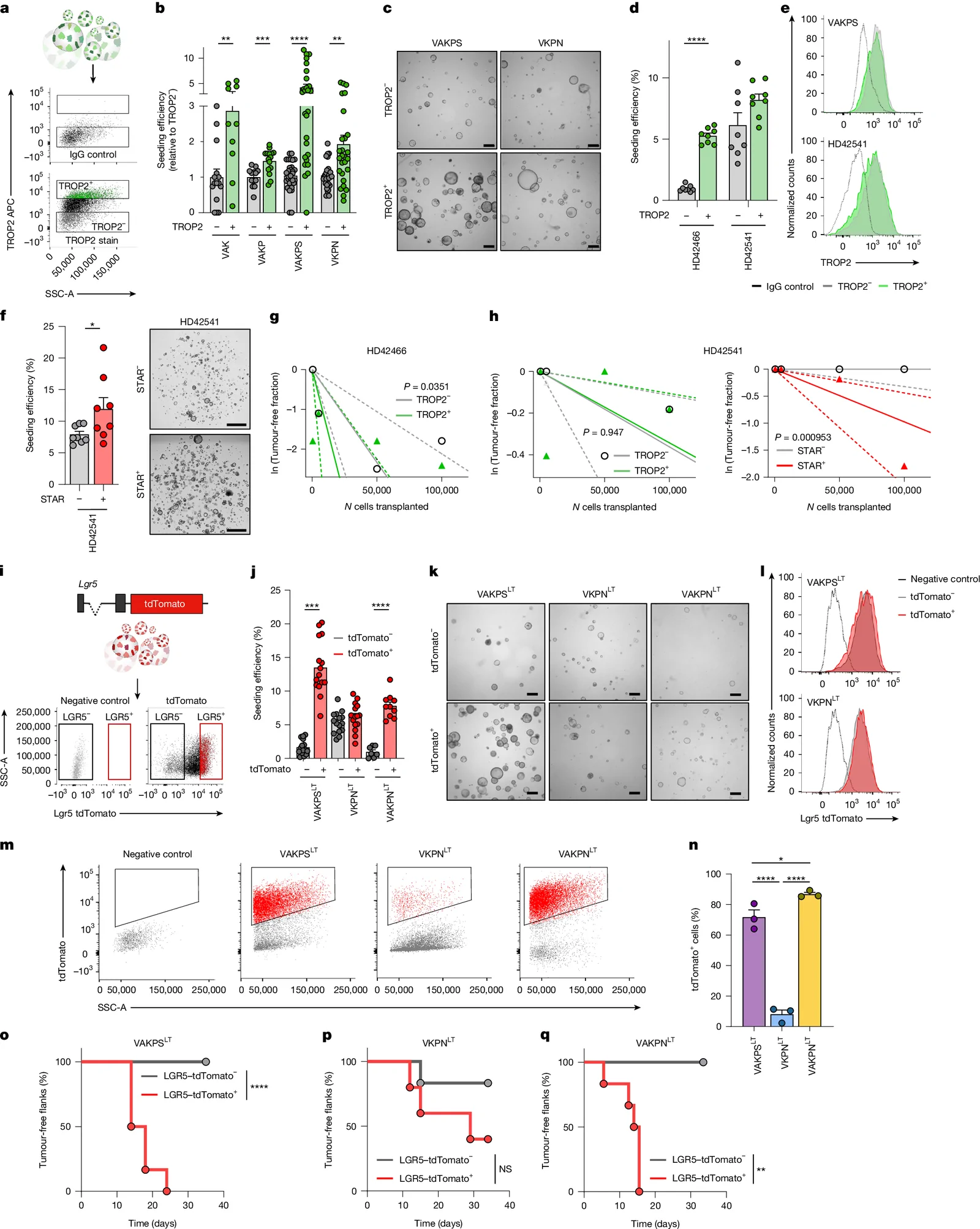
TROP2 marks CSCs of WNT^low^ CRC. **a**, Scheme of TROP2 cell population separation. **b**, Clonogenicity 5 days after seeding (*n* = 13, 15, 27 and 30 individual wells of up to five independent fluorescence-activated cell sorting (FACS) experiments). **c**, Images of organoid formation assays from **b**. **d**, Clonogenicity of PDO lines 10 days after seeding (HD42466, *n* = 8; HD42541, *n* = 8 individual wells of up to three independent FACS experiments). **e**, Histogram of TROP2^−^ and TROP2^+^ cells after seeding. Parental cell lines served as a negative control. **f**, Clonogenicity of PDO lines 10 days after seeding (HD42541, *n* = 8 individual wells from three independent FACS experiments). Right, images from organoid formation assays. **g**, In vivo limiting dilution assay (LDA) with TROP2^−^ or TROP2^+^ cells from HD42466 (*n* = 6 injections per dilution per group). *Y* axis represents the natural logarithm (ln) of tumour-free fraction. Stem cell frequency: 1 in 7,807 TROP2^+^ cells and 1 in 21,421 TROP2^−^ cells. **h**, In vivo LDA with TROP2^−^ or TROP2^+^ and STAR^+^ or STAR^−^ cells from HD42541 (*n* = 6 injections per dilution per group). *Y* axis represents the ln of tumour-free fraction. Stem cell frequency: 1 in 291,712 TROP2^+^ cells, 1 in 276,161 TROP2^−^ cells, 1 in 102,671 LGR5^+^ cells and undetectable in LGR5^−^ cells. **i**, Scheme of the tdTomato knock-in and FACS separation. **j**, Seeding efficiency of LGR5–tdTomato^+^ and LGR5–tdTomato^−^ cells 5 days after seeding (*n* = 15, 15 and 10 individual wells from three independent FACS experiments). **k**, Representative images of organoid formation assays from **j** 5 days after seeding. **l**, Histogram of the tdTomato signal 7 days after seeding; organoids derived from tdTomato^−^ and tdTomato^+^ cells. Parental cell lines served as a negative control. **m**, Dot plot of LGR5–tdTomato levels in CD45^−^CD31^−^EPCAM^+^ cells. **n**, tdTomato^+^ cell abundance across genotypes. Statistical analysis was performed using ordinary one-way ANOVA. **o**–**q**, Kaplan–Meier plots of genotypes (*n* = 6 mice per group) for VAKPS (**o**), VKPN (**p**) and VAKPN (**q**). Statistical analysis was performed using the log-rank (Mantel–Cox) test. In **b**, **d**, **f** and **j**, statistical analysis was performed using unpaired *t*-test. In **g** and **h**, statistical analysis was performed using a likelihood-ratio test. NS, nonsignificant. Scale bars, 100 µm (**c**,**k**), 1,000 µm (**f**). Schematics created in BioRender: **a**, Jackstadt, R. https://biorender.com/0xjr0wt (2026); **i**, Jackstadt, R. https://biorender.com/uzzv89x (2026).

**Extended Data Fig. 3:**
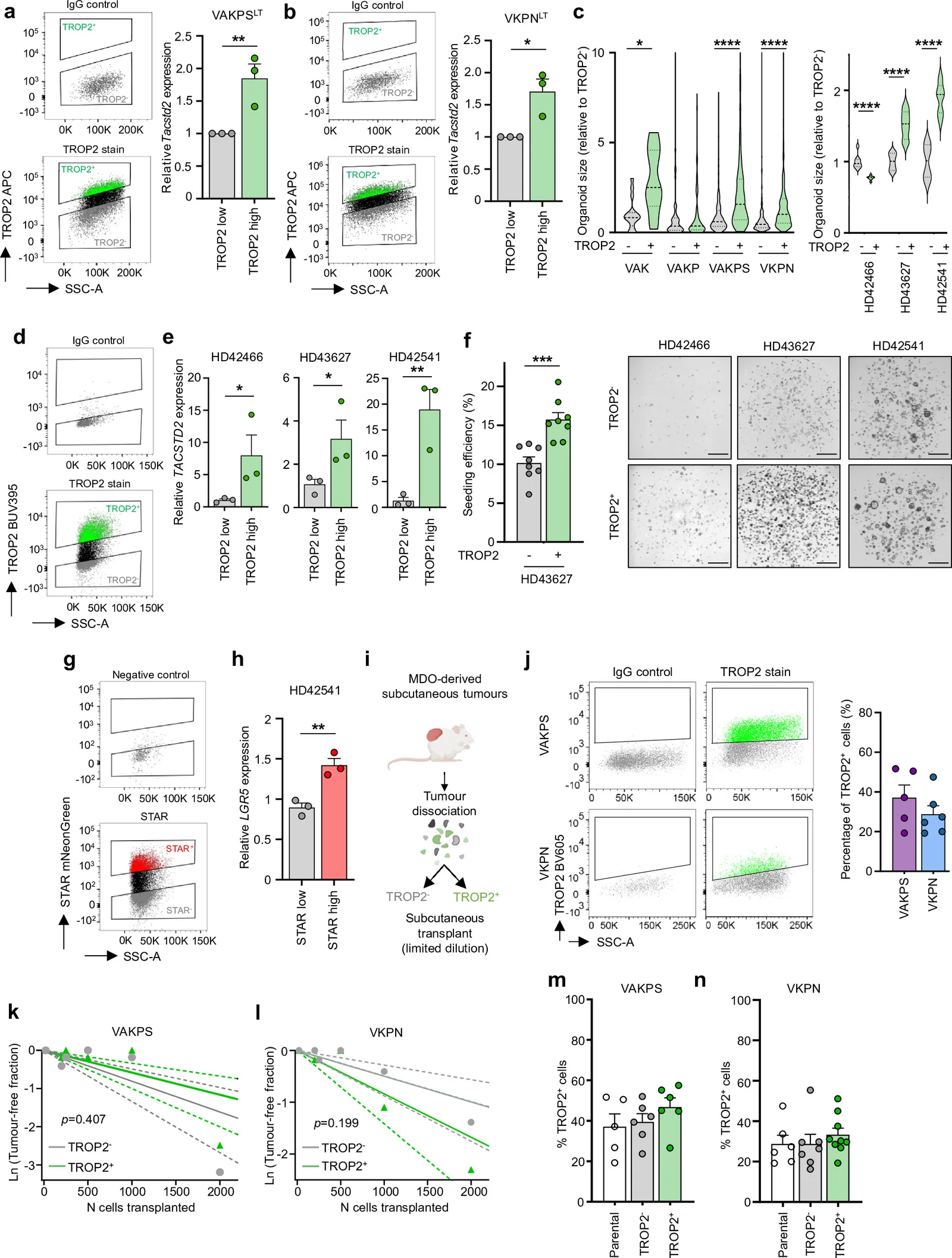
Characterisation of stem-like properties of TROP2^+^ cell. **a**, Representative FACS plots indicating the sorting strategy for TROP2 expressing cells. Quantitative RT-PCR of TROP2 sorted VAKPS^LT^ cells. Statistical analysis was performed using unpaired t-test. **b**, Representative FACS plots indicating the sorting strategy for TROP2 expressing cells. Quantitative RT-PCR of TROP2 sorted VKPN^LT^ cells. Statistical analysis was performed using unpaired t-test. **c**, MDO and PDO sizes normalized to mean size of TROP2^-^ respective organoids five days after seeding. Statistical analysis was performed using unpaired t-test. **d**, Representative FACS plots indicating the sorting strategy for TROP2 expressing cells. **e**, Quantitative RT-PCR of PDOs sorted for TROP2^+^ cells as indicated in **d**. Statistical analysis was performed using unpaired t-test. **f**, Clonogenicity of PDO lines ten days after seeding (HD43627, n = 8 individual wells from three independent FACS experiments). Statistical analysis was performed using unpaired t-test. Representative images from organoid formation assays from Fig. 2d. Scale bar 1000 µm. **g**, Representative FACS plots indicating the sorting strategy for STAR-mNeonGreen expressing PDOs. **h**, Quantitative RT-PCR of STAR-mNeonGreen sorted cells. Statistical analysis was performed using unpaired t-test. **i**, Scheme indicating the workflow for FACS separation of TROP2 cell populations. **j**, Representative flow cytometry dot plots of TROP2 levels in CD45^−^CD31^−^EPCAM^+^ cells from subcutaneous tumours. Quantification of TROP2^+^ cell abundance in CD45^−^CD31^−^EPCAM^+^ cells from subcutaneous tumours (n = 5, 6). **k**, and **l**, In vivo limiting dilution assay with indicated numbers of FACS separated TROP2^−^ or TROP2^+^ cells from VAKPS **k** and VKPN **l** tumours (n = 6-12 injections per dilution per group). Y-axis represents the natural logarithm (ln) of tumour-free fraction. LDA model fit (see methods) shown as solid line with 95% confidence intervals (dashed lines). Stem cell frequency VAKPS: 1/1721 (TROP2^+^), 1/1239 (TROP2^−^). Stem cell frequency VKPN: 1/1194 (TROP2^+^), 1/2012 (TROP2^−^). Statistical analysis was performed using a likelihood-ratio test. **m**, and **n**, Bar graph of TROP2^+^ tumour cells from tumours of **k-l**. Statistical analysis was performed using ordinary one-way ANOVA. Schematic in **i** created in BioRender; Jackstadt, R. https://biorender.com/q2ottvv (2026).

Next, we sought to investigate the functional role of TROP2^+^ cells in cancer stemness, a feature inherently associated with poor prognosis. Thus, we separated TROP2^−^ and TROP2^+^ (top 20%) cell populations and seeded them in vitro (Fig. 2a). In the aforementioned MDOs at various stages and in PDOs, TROP2^+^ cells demonstrated increased clonogenic capacity (Fig. 2b–d and Extended Data Fig. 3a–f). Notably, after 7 days in culture, organoids derived from TROP2^−^ or TROP2^+^ sorted populations displayed similar TROP2 levels, indicating a high degree of plasticity (Fig. 2e). PDOs with a reduced number of TROP2^+^ cells (HD42541) did not show significantly increased seeding capacity of TROP2^+^ cells in vitro (Fig. 2d). By contrast, when we labelled these PDOs with a WNT/LGR5 reporter (STAR)^34^, LGR5/WNT^hi^ cells showed increased seeding efficacy (Fig. 2f and Extended Data Fig. 3g,h). Next, we assessed the tumour-initiating capacities of TROP2 cells in vivo. To that end, we separated TROP2^−^ and TROP2^+^ tumour cells from MDOs or PDOs with low (HD42466/VKPN) and high (HD42541/VAKPS) WNT/LGR5 expression, followed by transplantation at serial dilutions. In the WNT^hi^ models, TROP2^+^ cells did not exhibit increased tumour-initiating capacities after transplantation, whereas in the WNT^low^ models TROP2^+^ cells displayed enhanced tumour-initiating capacity (Fig. 2g,h and Extended Data Fig. 3i–l). In both tumour types, high cellular plasticity led to a restoration of the TROP2^+^ cell pools over time (Extended Data Fig. 3m,n). These results highlight enhanced plasticity in CRC cells and suggest a potential dependency on the TROP2^+^ cell pool replenishment.

**Extended Data Fig. 4:**
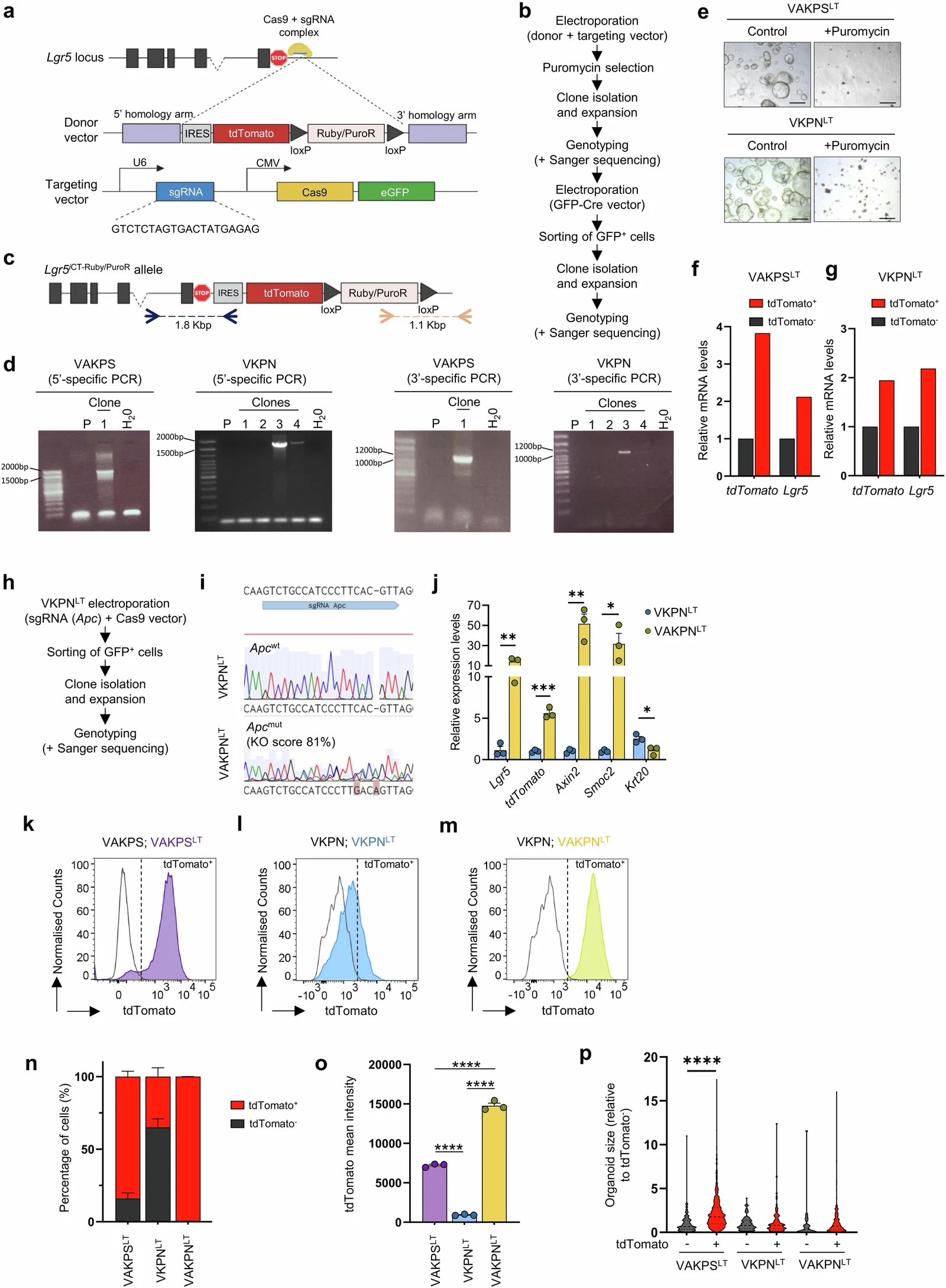
Genetic editing of MDOs. **a**, Scheme of the CRISPR/Cas9 strategy to knock-in the tdTomato cassette into the *Lgr5* locus. PuroR, Puromycin-resistant. **b**, Workflow for the generation of the MDO^LT^ lines. **c**, Scheme of the MDO^LT^ final allele. **d**, PCR product of the 5′ and 3′ specific PCRs for the generation of VAKPS^LT-Ruby/PuroR^ and VKPN^LT-Ruby/PuroR^ MDOs. **e**, Representative images of VAKPS^LT^ and KPN^LT^ MDOs four days after puromycin treatment. Scale bar 50 µm. **f**, and **g**, Quantitative PCR results showing *tdTomato* and *Lgr5* mRNA expression levels of tdTomato^-^ and tdTomato^+^ sorted cell populations. **h**, Workflow to generate *Apc* KO in VKPN^LT^ MDOs. **i**, Sanger sequence chromatogram confirming the gene editing of the *Apc* open reading frame. **j**, Quantitative PCR results of WNT-targets and differentiation markers. Statistical analysis was performed using unpaired t-test. **k**, **l**, and **m**, Flow cytometry histograms of tdTomato signal in **k** VAKPS parental and VAKPS^LT^ MDOs, **l** VKPN parental and VKPN^LT^ MDOs and **m** VKPN parental and VAKPN^LT^ MDOs. **n**, Relative abundance of tdTomato^+^ and tdTomato^-^ cells per MDO line (n = 3). **o**, Quantification of tdTomato signal intensity in the different MDO lines (n = 3). Statistical analysis was performed using ordinary one-way ANOVA. **p**, Quantification of organoid size from the clonogenicity assay five days after seeding. (VAKPS^LT^ n = 15, VKPN^LT^ n = 15, VAKPN^LT^ n = 10 individual wells from three independent experiments). Statistical analysis was performed using unpaired t-test. Schematics created in BioRender: **a**, Jackstadt, R. https://biorender.com/wyghbvw (2026); **c**, Jackstadt, R. https://biorender.com/wyghbvw (2026).

To define the stem cell capacity of LGR5-expressing cells in our CRC models, we knocked-in a tdTomato fluorescent reporter after the STOP codon of the *Lgr5* gene in VAKPS and VKPN MDOs (Fig. 2i and Extended Data Fig. 4a–e). Quantitative polymerase chain reaction (PCR) of isolated tdTomato^−^ and tdTomato^hi^ cells confirmed higher expression of *tdTomato* and *Lgr5* in tdTomato^hi^ cells, indicating selective labelling of *Lgr5*^*+*^ cells by tdTomato in hereafter called VAKPS^LT^ and VKPN^LT^ MDOs (Extended Data Fig. 4f,g). Separation of tdTomato^−^ and tdTomato^+^ (top 20%) cells revealed increased clonogenic potential in tdTomato^+^ VAKPS^LT^ cells. However, this difference was not detectable in WNT^low^ VKPN^LT^ MDOs (Fig. 2j,k). Querying whether this effect was WNT-dependent, we used CRISPR–Cas9 gene editing to delete *Apc* in VKPN^LT^ MDOs, hereafter VAKPN^LT^ MDOs. These MDOs showed significantly higher tdTomato and WNT-target gene expression compared with VKPN^LT^ MDOs (Extended Data Fig. 4h–o). The clonogenicity of tdTomato^+^ compared with tdTomato^−^ cells was also increased in VAKPN^LT^ MDOs (Fig. 2j,k and Extended Data Fig. 4p). Notably, the LGR5 population was quickly restored when plating tdTomato^−^ cells (Fig. 2l).

To further explore the stemness properties of the LGR5^+^ tumour cells in vivo, we separated the tdTomato^−^ and tdTomato^+^ tumour cells from subcutaneous tumours and transplanted them in the flank of secondary recipient mice (Fig. 2m–q). Similar to the in vitro data, tdTomato^+^ cells had higher tumour-initiating capacities than the tdTomato^−^ cells but only in *Apc*-deficient tumours (Fig. 2o–q). These findings suggest that LGR5 marks CSCs primarily in WNT-high (*Apc*-deficient) CRCs, whereas TROP2^+^ cells have stem-like tumour-initiating capacity in low WNT-signalling (*Apc*-proficient) CRCs.

## TROP2 defines metastasis-initiating cells

**Extended Data Fig. 5:**
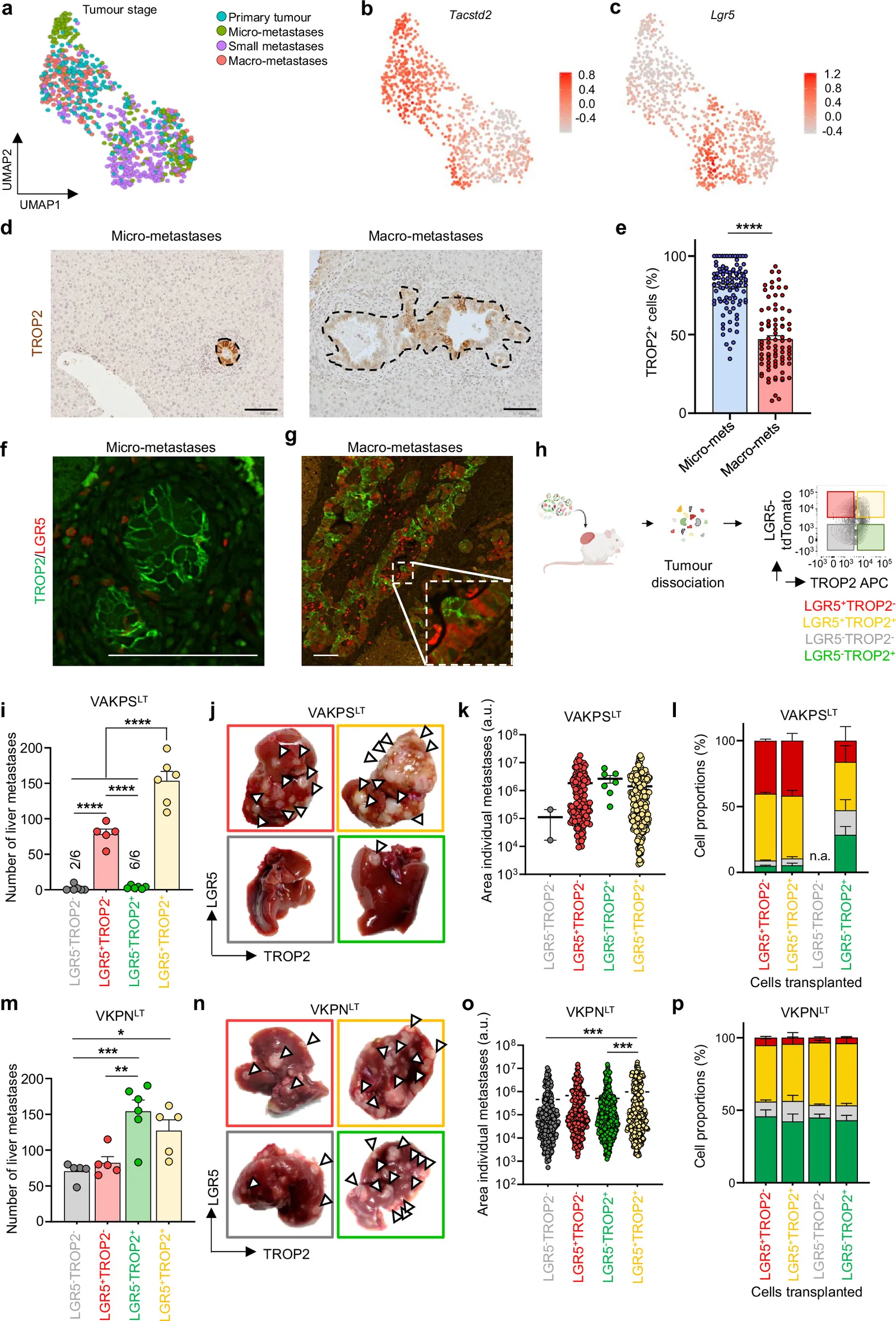
TROP2 in metastasis initiation and cellular plasticity. **a**, **b**, and **c**, UMAP layout from tumour cells (n = 900) coloured by **a** metastatic stage **b** *Tacstd2* and **c** *Lgr5* MAGIC-imputed expression levels. Data derived from^22^. **d**, Representative TROP2 IHC on micro- and macro-metastasis of AKP MDOs transplanted to C57BL/6 mice. Scale bar 100 µm. **e**, Quantification of TROP2-positive cells in micro and macro-metastasis (n = 4 mice for early stage and n = 5 mice for late stage). Statistical analysis was performed using unpaired t-test. **f-g**, Immunofluorescence of TROP2 (green) and LGR5-tdTomato (red) of VAKPS^LT^ liver **f** micro-metastases and **g** macro-metastases. Scale bar 100 µm. **h**, Scheme describing FACS separation of indicated cell populations derived from subcutaneous tumours for subsequent intra-splenic transplantation. The endpoint was 4 weeks post-injection. **i**, Bar graph showing the number of VAKPS^LT^ liver metastases originating from the different sorted cell populations in **h** (n = 5-6 per population). **j**, Representative images of livers from i. Arrows indicate metastatic nodules. **k**, Quantification of the cross-section area of the individual metastases derived from **j**. Mean and Standard Error of the Mean are plotted. **l**, Cell state composition of VAKPS^LT^ metastases stratified by seeding cell populations. n.a. = not assessed. **m**, Bar graph showing the number of VKPN^LT^ liver metastases originating from the different sorted cell populations in **h** (n = 5-6 per population). **n**, Representative images of the livers from m. Arrows indicate metastatic nodules. **o**, Quantification of the cross-section area of the individual metastases derived from **n**. Mean and Standard Error of the Mean is plotted. **p**, Cell state composition of VKPN^LT^ metastases stratified by seeding cell populations. Statistical analyses in **i**, **k**, **m**, **o** were performed using ordinary one-way ANOVA. Schematic in **h** created in BioRender; Jackstadt, R. https://biorender.com/39vd9js (2026).

Metastasis-initiating cells (MICs) and micro-metastases represent a highly patient-relevant stage in CRC progression. Although loss of LGR5 was already observed at the tumour invasion front, a fetal-like state has been described as being critical for metastatic progression^17,24^. To define the relevance of TROP2^+^ cells in metastatic colonization and to interrogate the functional plasticity between cell states, we analysed the expression of TROP2 across metastatic stages ranging from micro-metastasis to small metastasis and macro-metastasis^22^ (Extended Data Fig. 5a–c). We detected LGR5 expression in larger lesions, whereas TROP2 was already present at the initial event of metastatic seeding (Extended Data Fig. 5a–g). To test the relevance of TROP2^+^ cells during metastasis formation, we separated tumour cells according to their TROP2 and LGR5 levels from VAKPS^LT^ and VKPN^LT^ tumours (Extended Data Fig. 5h). In a metastatic seeding assay, LGR5^+^ sorted cells of VAKPS^LT^ tumours had a significantly higher metastatic capacity than the LGR5^−^ cells (Extended Data Fig. 5h–k). Nevertheless, when we compared the potential of LGR5^+^/TROP2^−^ and LGR5^+^/TROP2^+^ cells to generate macro-metastases, double-positive cells formed significantly more metastases (Extended Data Fig. 5h–k). Furthermore, when we assessed the metastatic capacity of LGR5^−^ cells, previously defined as MICs^17^, we detected a significant increase in metastatic efficacy for TROP2^+^ cells (Extended Data Fig. 5i–l). In VKPN^LT^ MDOs, both TROP2^+^ sorted cell populations had significantly higher metastatic capacity than the TROP2^−^ cell populations regardless of LGR5 levels (Extended Data Fig. 5m–o), in line with their increased seeding efficiency in vitro. Together, TROP2^+^ cells have a higher metastatic capacity than TROP2^−^ cells in both VAKPS and VKPN models. Additionally, all generated metastases swiftly restored a balanced cell-state equilibrium, demonstrating high plasticity of CRC cells (Extended Data Fig. 5l,p).

## TROP2 targeting and cell state remodelling

**Fig. 3:**
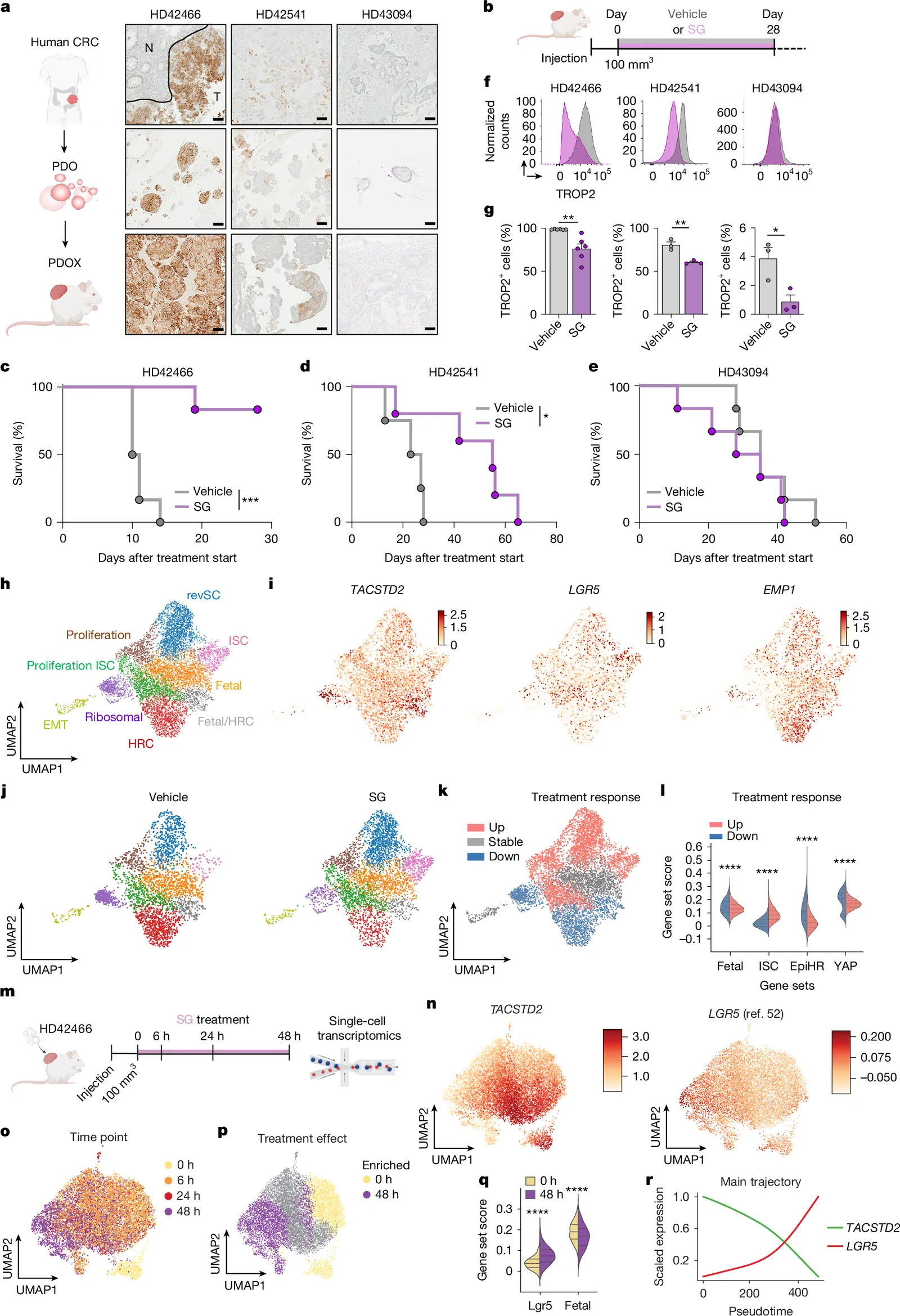
**Targeting TROP2 remodels cancer cell states.** **a**, Immunohistochemistry of TROP2 in patient samples, PDOs and PDOXs. **b**, Scheme indicating the treatment regimen with vehicle or SG. **c**–**e**, Kaplan–Meier plots of survival of TROP2^hi^ (HD42466, *n* = 6 and 6) (**c**), TROP2^medium^ (HD42541, *n* = 4 and 5) (**d**) and TROP2^low^ (HD43094, *n* = 6 and 6) (**e**) PDOXs after treatment with SG, as shown in **b**. Statistical analysis was performed using the log-rank (Mantel–Cox) test. **f**, Representative flow cytometry histogram of TROP2 levels after SG treatment in PDOXs (**c**–**e**). **g**, Abundance of TROP2^+^ cells upon SG treatment in TROP2^hi^ (*n* = 6 and 6), TROP2^medium^ (*n* = 3 and 3) and TROP2^low^ (*n* = 3 and 3) PDOXs (**c**–**e**). Statistical analysis was performed using unpaired *t*-test. **h**, UMAP embedding with annotated tumour cell states after treatment of HD42466 PDOXs with vehicle or SG from **c** (*n* = 7,330 cells; four tumours per treatment). **i**, UMAP representation of marker gene expression levels. Colour scale up to 99th percentile. **j**, UMAP as shown in **h** (matching colours) split by treatment condition. **k**, UMAP showing clusters increasing or decreasing upon treatment with SG. **l**, Violin plots of gene set scores stratified by cluster-based treatment response shown in **k** with dashed lines indicating quartiles. Distributions were compared using a Wilcoxon rank-sum test with Benjamini–Hochberg correction for multiple testing. **m**, Scheme of in vivo SG metastasis treatment and sampling time points. **n**, UMAP representation of data in **m** showing *TACSTD2* imputed expression values (up to 99.5th percentile) and ISC LGR5 gene set score (*n* = 11,385 cells; one to two mice per time point). **o**, UMAP colour-coded by time point. **p**, UMAP colour-coded cluster size differences between 0 h and 48 h time point. **q**, Violin plot showing gene set score changes over time with dashed lines indicating quartiles. Distributions were compared using a Wilcoxon rank-sum test with Benjamini–Hochberg correction for multiple testing. **r**, Normalized gene expression changes (min–max scaled) along pseudotime of main Palantir trajectory. N, normal; T, tumour. Scale bar, 100 µm (**a**). Schematics created in BioRender:** a**, Jackstadt, R. https://biorender.com/umdaj9r (2026); **b**, Jackstadt, R. https://biorender.com/3w435x2 (2026); **m**, Jackstadt, R. https://biorender.com/29u0lf3 (2026).

**Extended Data Fig. 6:**
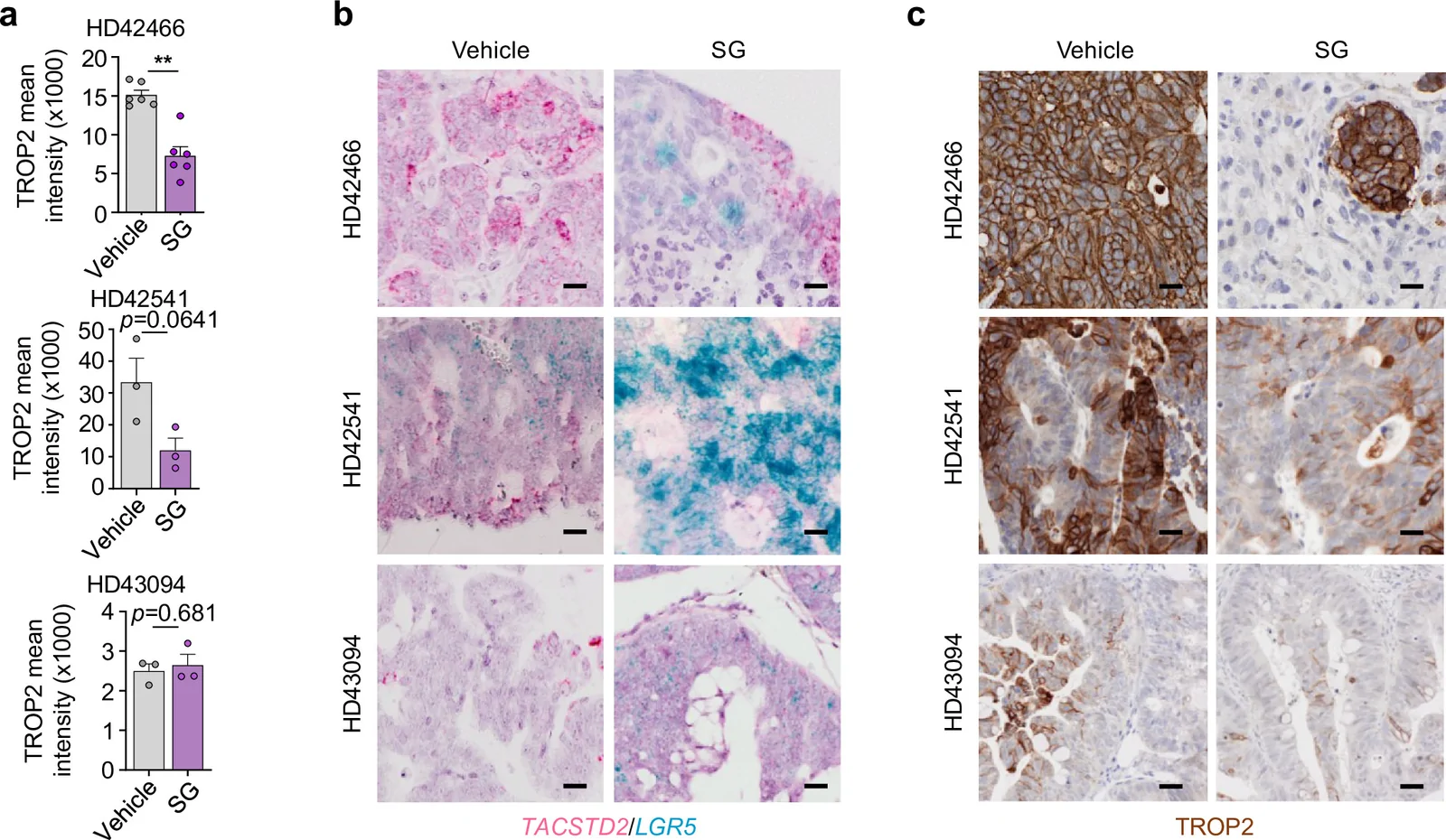
Cell state analysis upon TROP2 cell targeting. **a**, Quantification of the TROP2 mean intensity upon SG treatment in TROP2-high (n = 6, 6), TROP2-medium (n = 3, 3) and TROP2-low (n = 3, 3) PDOXs related to Fig. 3c–e. Statistical analysis was performed using unpaired t-test. **b**, In situ hybridisation of *TACSTD2* and *LGR5* on treated PDOXs related to Fig. 3c–e. Scale bar 50 µm. **c**, Immunohistochemistry of TROP2 on treated PDOXs related to Fig. 3c–e. Scale bar 50 µm.

**Extended Data Fig. 7:**
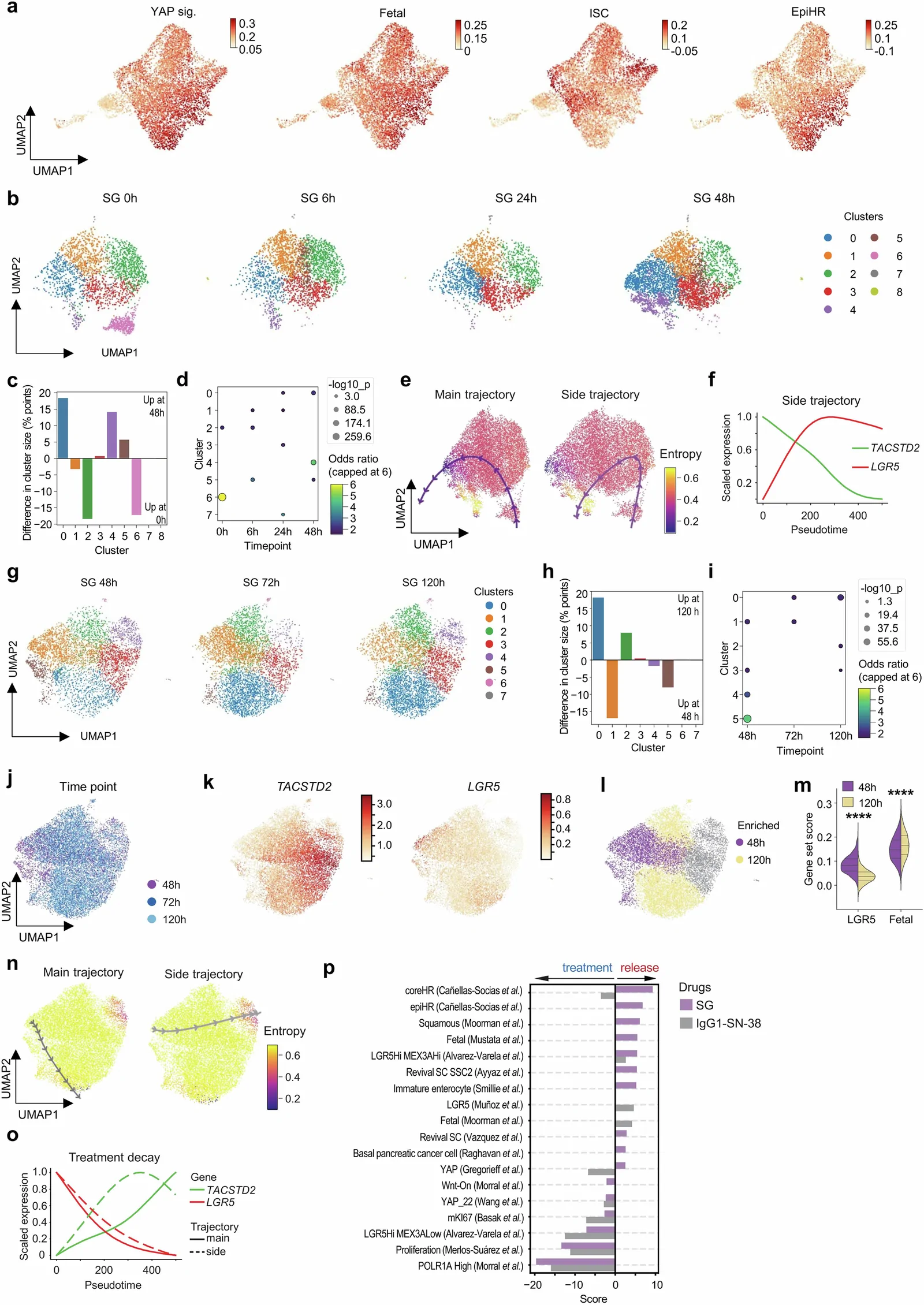
Cell state dynamics upon TROP2 cell targeting. **a**, UMAP representation of imputed gene expression values (saturated at 99th percentile). Related to Fig. 3h. **b**, UMAP with leiden clustering split by time point with on-treatment effects. Related to Fig. 3m–r. **c**, Difference in relative cluster size at indicated time points. **d**, Odds ratio of enrichment of time points in clusters using Fisher’s exact test. (Odds ratio capped at 6 for visualisation). **e**, Inferred palantir trajectories on entropy UMAPs. The main trajectory represents the temporal distribution changes depicted in **b-d**. **f**, Gene expression changes (min-max scaled) along pseudotime for side trajectory. **g**, UMAP with leiden clustering split by time point with drug waning effects (n = 13,984 cells, 2 mice per time point). **h**, Difference in relative cluster size at indicated time points. **i**, Odds ratio of enrichment of time points in clusters using Fisher’s exact test. (Odds ratio capped at 6 for visualisation). **j**, UMAP colour-coded by time point. **k**, UMAP representing imputed expression values of *TACSTD2* and *LGR5*. **l**, UMAP colour-coded by cluster size differences at 48 h and 120 h. **m**, Violin plot showing gene set score changes over time with dashed lines indicating quartiles. (Statistic: Wilcoxon rank sum test with Benjamini Hochberg correction for multiple testing). **n**, Inferred palantir trajectories on entropy UMAP. The main trajectory represents distribution changes depicted in **g**-**i**. **o**, Gene expression changes (min-max scaled) along pseudotime for both trajectories. **p**, Gene set enrichment analysis results using a univariate linear model based on pseudobulk DGEA between drug treatment and release conditions (p-adj <0.05). Cells were sampled during treatment with either SG (purple) or untargeted ADC (IgG1-SN-38, grey) for up to 12 h (treatment). After drug washout (release), additional sampling was performed up to 72 h (see methods). A total of 23,788 cells and 23,118 cells were analysed for SG and IgG1-SN-38 treatment, respectively, based on one sample for each of the narrowly-spaced time steps.

Our results show that TROP2-expressing cells are associated with tumour cell populations linked to poor clinical outcomes. Hence, targeting these cells bears high clinical relevance. In recent years, the use of TROP2-targeting ADCs, such as SG, has shown striking efficacy and clinical success in triple-negative breast cancer^35^. Hence, we aimed to target TROP2-expressing cells in CRC. We used our biobank of patient-derived CRC samples, which showed heterogeneous TROP2 expression (Fig. 3a). PDOs and PDOXs generated from these samples recapitulated the expression and membranous localization of TROP2 found in primary tissue (Fig. 3a). Treatment of established PDOXs with SG highlighted a TROP2 expression-dependent survival benefit (Fig. 3b–e). This benefit was accompanied by reduction of TROP2 cell-surface protein levels (Fig. 3f,g and Extended Data Fig. 6a). Next, we considered that TROP2^+^ cell targeting might remodel the cellular state composition of tumours. Single-cell transcriptomics of SG-treated and vehicle-treated PDOX tumours revealed the existence of nine epithelial clusters (Fig. 3h). As previously detected, we identified a composition of ISCs (marked by *LGR5*), revival stem cells (marked by *CLU*), fetal-like cells (marked by *TACSTD2*) or HRCs (marked by *EMP1*) (Fig. 3h,i). Further analysis showed that SG treatment was linked to a marked reduction in HRC-like and fetal-like signatures and increased ISC/LGR5 signatures (Fig. 3j–l and Extended Data Figs. 6b,c and 7a). To resolve the temporal dynamics of this, we next analysed PDOs of an in vivo treatment time-course experiment (Fig. 3m). A single dose of SG led to a clear change in cell-state composition during the first 48 h, leading to an upregulation of the LGR5 program at the expense of TROP2^+^ and fetal-like cells (Fig. 3n–q and Extended Data Fig. 7b–d). Trajectory inference with Palantir further shows a shift from a TROP2^hi^ state towards an ISC-like LGR5 cell state during SG treatment (Fig. 3r and Extended Data Fig. 7e,f). This cell state shift was reversible when the drug effect waned^36,37^ (Extended Data Fig. 7g–o). To validate this further, we used an in vitro time course of drug treatment followed by drug washout using both SG and an untargeted ADC control with identical payload IgG1–SN-38. SG treatment showed an upregulation of the WNT program under treatment, followed by pronounced reappearance of HRC-like, non-canonical-like and fetal-like programs upon drug release (Extended Data Fig. 7p). This plastic change in gene expression programs was not observed with the untargeted ADC, which instead presented mainly with the emergence of LGR5 signatures upon drug release. This provides compelling evidence for TROP2-targeted SG treatment dynamics. Together, we demonstrate that targeting TROP2^+^ cells causes a state remodelling towards ISC states.

## Chemotherapy-induced TROP2 and plasticity

**Fig. 4:**
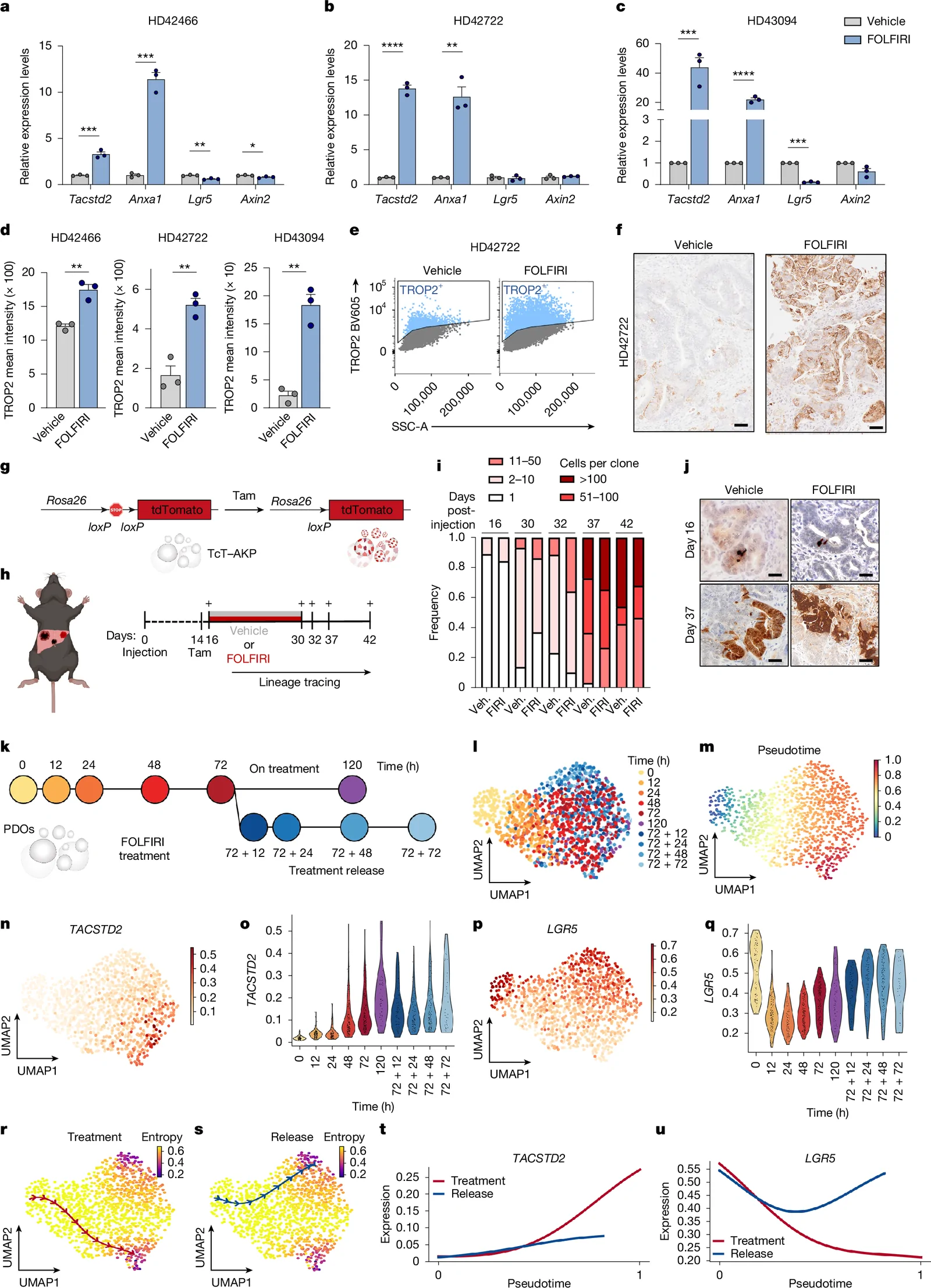
**Chemotherapy-induced TROP2 and plasticity.** **a**–**c**, Quantitative PCR results of PDOs treated with vehicle or FOLFIRI for 96 h for lines HD42466 (**a**, *n* = 3), HD42722 (**b**, *n* = 3) and HD43094 (**c**, *n* = 3). **d**, Quantification of TROP2 mean intensity in different PDOs treated with vehicle or FOLFIRI for 96 h. **e**, Representative flow cytometry dot plots from **d**. **f**, Immunohistochemistry of TROP2 from PDOXs treated with vehicle or FOLFIRI for 4 days. **g**, Scheme indicating recombination after 4-OHT application. **h**, Illustration of the lineage-tracing approach in TcT–AKP MDO liver metastasis. **i**, Quantification of tdTomato-labelled clone size as described in **h** (*n* = 5–6 mice per condition; *n* = 18, 19, 44, 65, 26, 50, 33, 49, 26 and 28 analysed clones). **j**, Representative image of tdTomato-labelled clones in liver metastasis. **k**, Schematic indicating the treatment regimen of PDOs (HD43478) with FOLFIRI. **l**, UMAP representing the time points of treatment and release (*n* = 1,173 cells; one replicate per time point). **m**, UMAP representing the Palantir pseudotime. **n**, UMAP representing the imputed expression levels of *TACSTD2*. **o**, Violin plots of imputed *TACSTD2* expression levels over time. **p**, UMAP representing the imputed expression levels of *LGR5*. **q**, Violin plots of imputed *LGR5* expression over time. **r**,**s** Treatment (**r**) and release trajectory (**s**) on entropy UMAP. **t**, *TACSTD2* expression over pseudotime along trajectories (**r**,**s**). **u**, *LGR5* expression over pseudotime along trajectories (**r**,**s**). In **a**–**d**, statistical analysis was performed using unpaired *t*-tests. Veh., vehicle. Scale bars, 50 µm (**f**), 100 µm (**j**). Schematics created in BioRender: **g**, Jackstadt, R. https://biorender.com/vqdkg63 (2026);** h**, Jackstadt, R. https://biorender.com/8psxkns (2026); **k**, Jackstadt, R. https://biorender.com/3w435x2 (2026).

**Extended Data Fig. 8:**
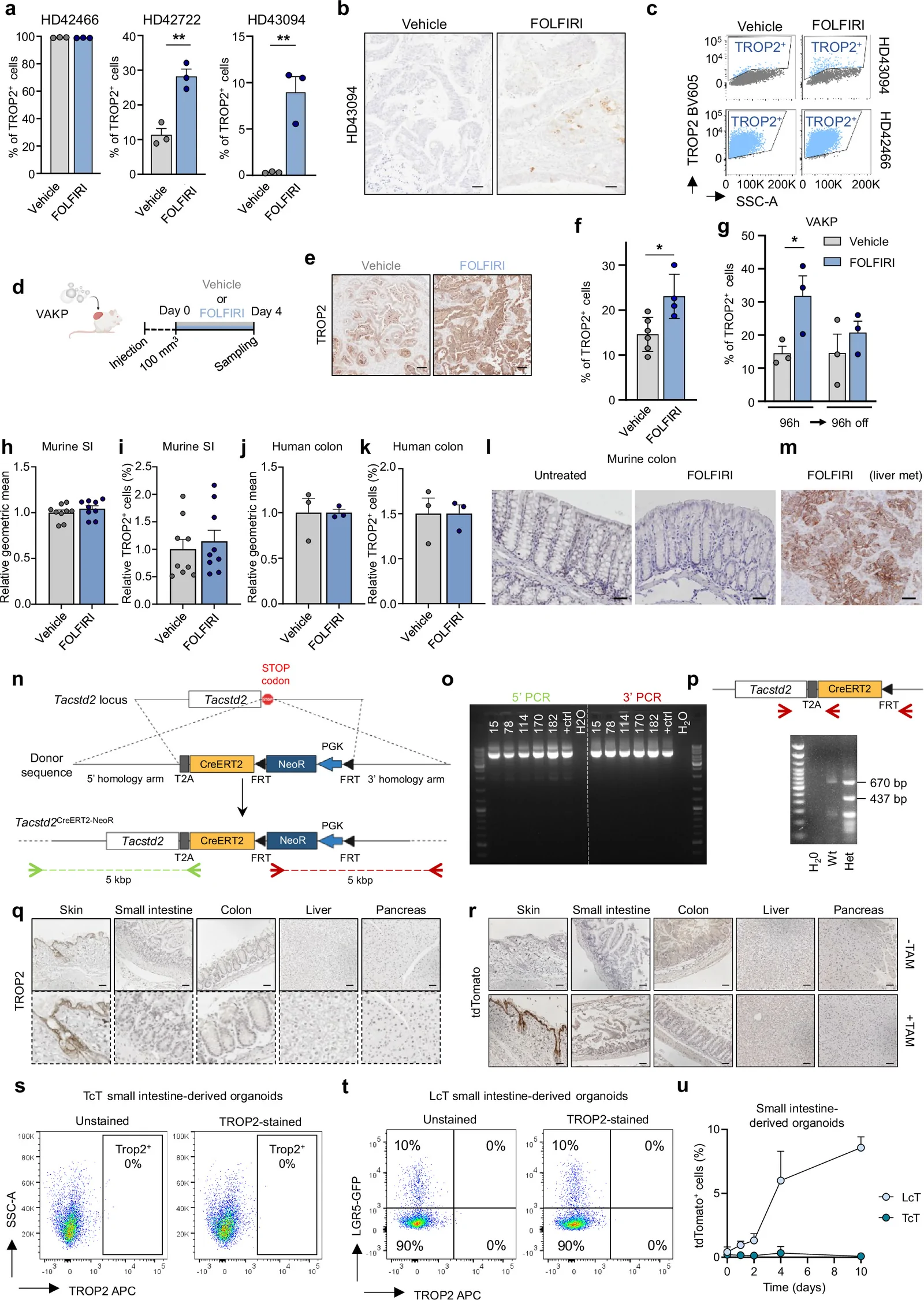
Generation and validation of the *Tacstd2*^CreER^ transgene. **a**, Quantification of TROP2^+^ cell abundance in PDOs treated with FOLFIRI or vehicle for 96 h. **b**, Immunohistochemistry of TROP2 from PDOXs treated with vehicle or FOLFIRI for 4 days. Scale bar 50 µm. **c**, Representative flow cytometry dot plots from **a**. **d**, Scheme indicating the treatment regimen of subcutaneously transplanted VAKP MDOs into C57BL/6 mice. **e**, Representative TROP2 immunohistochemistry of tumours from **d**. **f**, Quantification of TROP2^+^ cells as measured by flow cytometry in VAKP tumours four days after treatment from **d**. **g**, Quantification of TROP2^+^ cells as measured by flow cytometry in VAKP MDOs 96 h after treatment followed by 96 h of drug release. Statistical analysis was performed using Mann-Whitney U test. **h**, Quantification of mean intensity of TROP2 in normal small intestinal (SI) mouse organoids 72 h after FOLFIRI or vehicle treatment. Technical replicates from three individual experiments. **i**, Quantification of TROP2^+^ cell abundance in normal small intestinal (SI) mouse organoids 72 h after FOLFIRI or vehicle treatment. Technical replicates from three individual experiments. **j**, Quantification of mean intensity of TROP2 in normal colon human organoids 96 h after FOLFIRI or vehicle treatment. Each data point represents two technical replicates from three individual donors. **k**, Quantification of TROP2^+^ cells in normal colon human organoids 96 h after FOLFIRI or vehicle treatment. Each data point represents two technical replicates from three individual donors. **l**, Representative IHC of TROP2 stain in colonic mucosa of untreated or FOLFIRI treated C57BL/6 mice. Scale bar 200 µm. **m**, Representative IHC of FOLFIRI treated liver metastases as control for **l**. Scale bar 200 µm. **n**, Scheme illustrating the *Tacstd2* gene targeting strategy to knock-in the “T2A-CreERT2-FRT-NeoR-FRT” cassette. The green and red arrows indicate the annealing site of the primers for the 5′ and 3′ specific PCR, respectively. **o**, PCR products of the five ES colonies with transgene integration. **p**, Scheme illustrating the genotyping strategy and PCR product of the *Tacstd2*^CreERT2^ allele (Wt=wild-type, het=heterozygous). **q**, Representative immunohistochemistry images of TROP2 across different murine tissues. Scale bar 50 µm. Dashed lines indicate higher magnification. **r**, Representative images of tdTomato immunohistochemistry six days after tamoxifen induction in *Tacstd2*^CreERT2^ LSL-tdTomato (TcT) mice. Scale bar 50 µm. **s**, Flow cytometry plot for TROP2 in TcT small intestine-derived organoids. **t**, Flow cytometry plot showing the percentage of TROP2^+^ and LGR5-GFP^+^ cells in *Lgr5*^eGFP-CreERT2^ LSL-tdTomato (LcT) small intestine derived organoids. **u**, Quantification of flow cytometry data of tdTomato^+^ cells in TcT and LcT small intestine-derived organoids at different days after 4-OHT pulse. Frequency of populations refers to the number of viable cells. Statistical analysis in **a**, **f**, **g**, **h**, **i**, **j**, **k** was performed using unpaired t-test. Schematics created in BioRender: **d**, Jackstadt, R. https://BioRender.com/3w435x2 (2026); **n**, Jackstadt, R. https://biorender.com/bestont (2026); **p**, Jackstadt, R. https://biorender.com/gxq9i3e (2026).

Next, we sought to define mechanisms that may drive TROP2 expression in a setting that is clinically relevant with the goal of optimizing TROP2-targeting therapies to benefit a wider spectrum of patients. Previous reports demonstrate that fetal-like progenitor signatures are induced upon chemotherapy treatment^19,20,24,38^. When we treated various PDOs with FOLFIRI, we observed an induction of TROP2 expression, independent of baseline TROP2 levels in all models (Fig. 4a–f and Extended Data Fig. 8a–c). Even in tumours in which every cell expressed TROP2 at baseline, we found significantly enhanced TROP2 surface levels upon FOLFIRI exposure (Fig. 4d and Extended Data Fig. 8a). TROP2 induction was also detected upon FOLFIRI exposure of VAKP MDOs in vitro and in vivo (Extended Data Fig. 8d–f). This induction was reverted 96 h after drug withdrawal, indicating a high degree of plasticity (Extended Data Fig. 8g). Notably, the same FOLFIRI treatment of mouse or human normal tissue organoids and of mouse colon tissue in vivo did not show TROP2 induction (Extended Data Fig. 8h–m).

To define whether TROP2 expression is induced upon chemotherapy or whether TROP2-expressing cells were selectively enriched, we generated a *Tacstd2*^*CreER*^ allele by introducing a CreER open reading frame in the 5′ untranslated region (5′UTR) of the *Tacstd2* gene linked with a T2A site (Extended Data Fig. 8n–p). We introduced the *Tacstd2*^*CreER*^ allele to LSL–tdTomato mice (TcT). Functional validation of the allele showed that only skin, in which TROP2 is expressed under homeostatic conditions, led to tdTomato labelling after tamoxifen injection, whereas small intestine, colon, liver and pancreas remained negative (Extended Data Fig. 8q,r). Additionally, we generated normal intestine-derived organoids and assessed the tdTomato labelling after 4-hydroxy-tamoxifen (4-OHT) addition (Extended Data Fig. 8s–u). After 10 days, the TcT allele did not show any tdTomato-labelled cells, whereas *Lgr5*^*GFP-CreER*^ *Rosa26*^*LSL-tdTomato*^ small intestinal organoids (LcT) showed efficient labelling (Extended Data Fig. 8u), demonstrating the lack of TROP2 expression in normal tissues.

**Extended Data Fig. 9:**
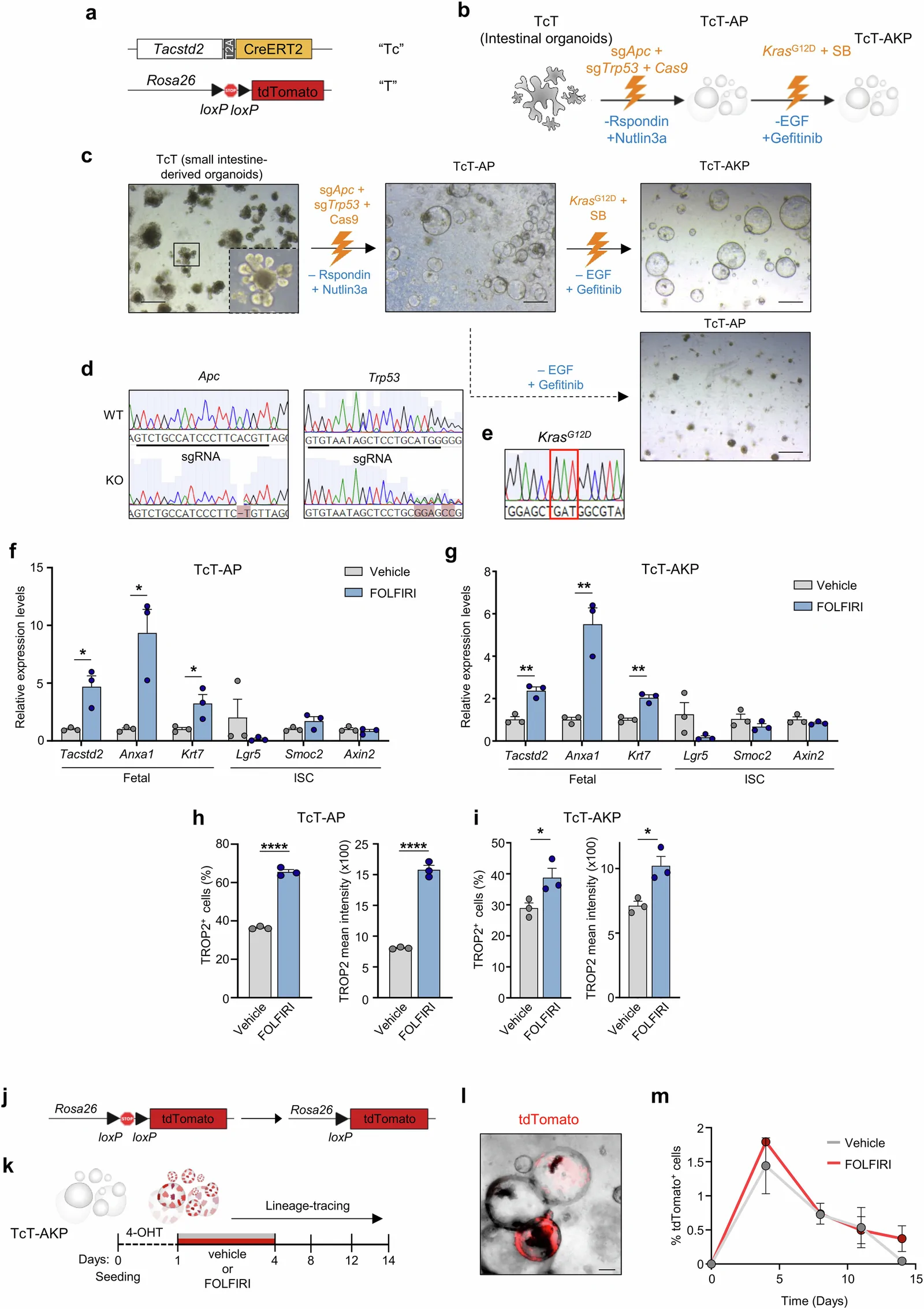
Chemotherapy induced TROP2 expression. **a**, Scheme of the genetic crossing strategy to generate TcT GEMMs for lineage tracing. **b**, Scheme describing the generation of *Tacstd2*^CreERT2^, LSL-tdTomato, *Apc*-KO, *Trp53*-KO (TcT-AP) and *Tacstd2*^CreERT2^, LSL-tdTomato, *Apc*-KO, *Kras*^G12D^ *Trp53*-KO (TcT-AKP) MDOs utilising CRISPR/Cas9 genome editing and the sleeping beauty (SB) systems. **c**, Representative images of TcT organoids after selection with reduced media. Scale bar 200 µm. **d**, Sanger sequence chromatogram of gene editing result. **e**, Sanger sequence chromatogram of transgene insertion. **f**, Quantification of RT-qPCR results of TcT-AP MDOs treated with FOLFIRI or vehicle for 96 h. **g**, Quantification of RT-qPCR results of TcT-AKP MDOs treated with FOLFIRI or vehicle for 96 h. **h**, Quantification of TROP2^+^ cell abundance and TROP2 mean intensity in TcT-AP MDOs treated with FOLFIRI or vehicle for 96 h. **i**, Quantification of TROP2^+^ cell abundance and TROP2 mean intensity in TcT-AKP MDOs treated with FOLFIRI or vehicle for 96 h. **j**, Scheme indicating the recombination after 4-OHT application. **k**, Illustration describing the lineage-tracing approach in TcT-AKP MDOs. **l**, Representative picture of tdTomato labelled organoids. Scale bar 100 µm. **m**, Quantification of tdTomato labelled cell abundance. Statistical analysis in **f**, **g**, **h**, **i** was performed using unpaired t-test. Schematics created in BioRender:** a**, Jackstadt, R. https://biorender.com/vqdkg63 (2026);** b**, Jackstadt, R. https://biorender.com/11hokdk (2026); **c**, Jackstadt, R. https://biorender.com/11hokdk (2026); **j**, Jackstadt, R. https://biorender.com/vqdkg63 (2026); **k**, Jackstadt, R. https://biorender.com/x9hy3nb (2026).

**Extended Data Fig. 10:**
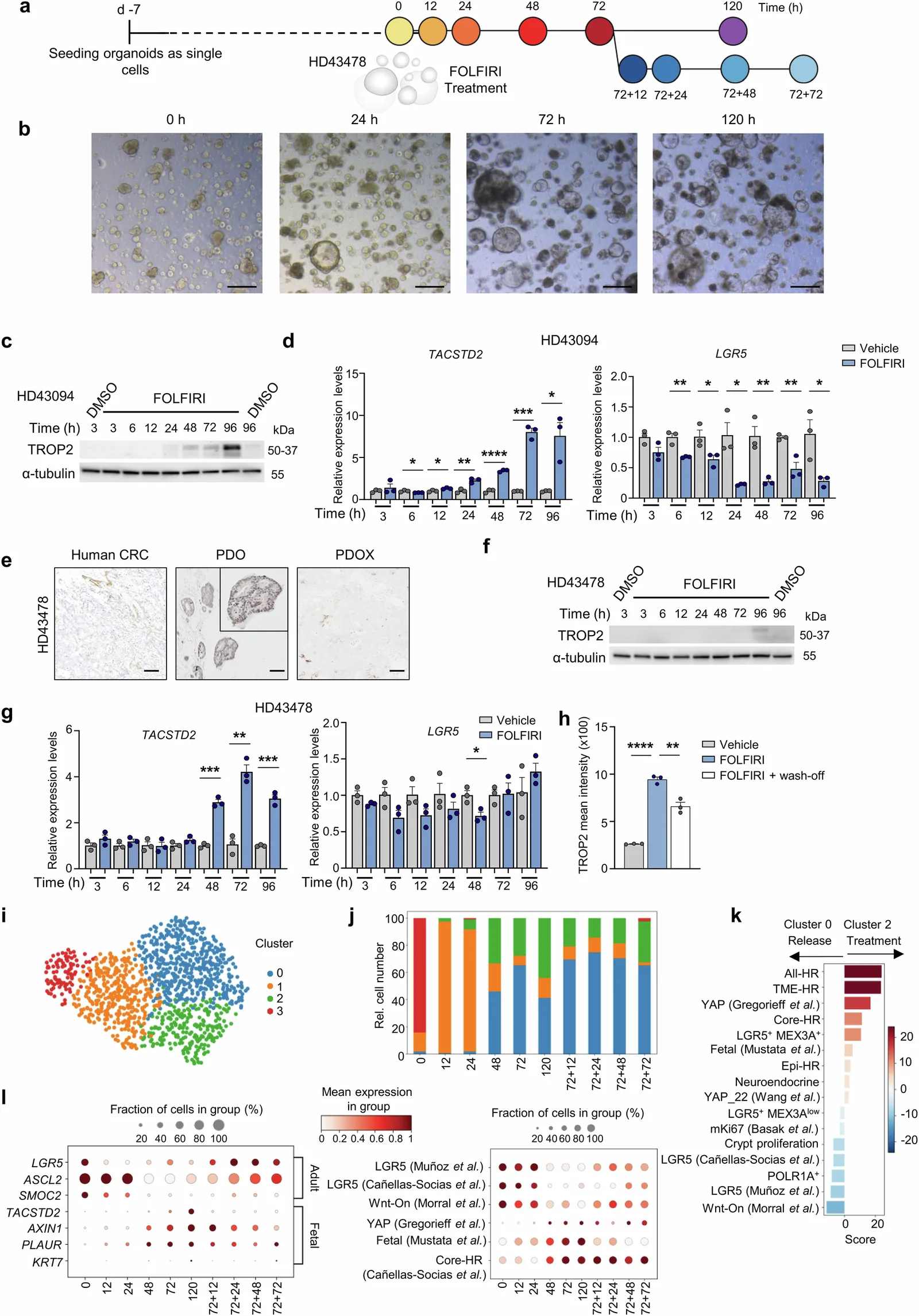
Cell state dynamics upon chemotherapy treatment. **a**, Schematic indicating the treatment regimen of PDOs with FOLFIRI and release indicated in blue. **b**, Microscopy pictures of PDOs at indicated time points as described in **a**. Scale bar 200 µm. **c**, Western blot of indicated proteins after FOLFIRI treatment. **d**, Quantitative PCR analysis of *TACSTD2* or *LGR5* expression at indicated time points of treatment with FOLFIRI or vehicle (n = 3 technical replicates per condition). **e**, Representative pictures of TROP2 IHC in human primary tumour and matched PDOs or PDOXs. Scale bar 100 µm. **f**, Western blot of indicated proteins after FOLFIRI treatment. **g**, Quantitative PCR analysis of *TACSTD2* or *LGR5* expression in indicated time points of treatment with FOLFIRI or vehicle (n = 3). **h**, Quantification of TROP2 mean intensity in PDOs (HD43478) treated with FOLFIRI or vehicle for 72 h or 72 h after the drug release. **i**, UMAP embedding of leiden clusters, related to Fig. 4k–u. **j**, Bar graph of relative cluster size over treatment and release time points of i. **k**, Enrichment analysis of public gene sets using a univariate linear model based on differentially expressed genes between terminal “on-treatment” cluster 2 and “release” cluster 0. Significant results (p-adj <0.05) are shown. **l**, Bubble plot of scaled marker gene expression at indicated time points. Schematic in **a** created in BioRender; Jackstadt, R. https://biorender.com/3w435x2 (2026). Statistical analyses in **d**, **g**, **h** were performed using unpaired t-test.

To transform these organoids, we used CRISPR–Cas9-mediated gene editing to knockout *Apc* and *Trp53* (TcT–AP) and introduced the most prevalent *Kras* (G12D) mutation in CRC (TcT–AKP) (Extended Data Fig. 9a–e). We next characterized the response of TcT–AP and TcT–AKP MDOs to FOLFIRI treatment. This treatment induced TROP2 expression on mRNA and protein levels in both genotypes, along with the fetal-like markers *Krt7* and *Anxa1* (Extended Data Fig. 9f–i). By contrast, *Lgr5* expression was reduced (Extended Data Fig. 9f,g). To test whether FOLFIRI treatment leads to a persister cell selection, we injected the generated TcT–AKP MDOs intrasplenically and lineage-traced the clonal expansion in the emerging liver metastases (Fig. 4g–j and Extended Data Fig. 9j–m). Longitudinal analysis of the tdTomato clones in vitro and in vivo revealed no apparent differences between treatment groups (Fig. 4i and Extended Data Fig. 9m), suggesting an upregulation of TROP2 levels by chemotherapy. To dissect the dynamics of the transcriptional cell state remodelling, we performed longitudinal treatment analysis in PDOs (Fig. 4k,l and Extended Data Fig. 10a,b). We detected a time-dependent increase in TROP2 protein and mRNA, along with downregulation of *LGR5* (Fig. 4m–q and Extended Data Fig. 10c–l). Trajectory inference and pseudotime analysis suggests a FOLFIRI-induced increase of the TROP2^hi^ state, whereas drug release uncovers a reversion to a LGR5/WNT^hi^ cell state (Fig. 4r–u and Extended Data Fig. 10k,l). In sum, we show that chemotherapy induces TROP2 expression, accompanied by a therapy-associated change in tumour cell states.

## Combination therapy enhances efficacy

**Fig. 5:**
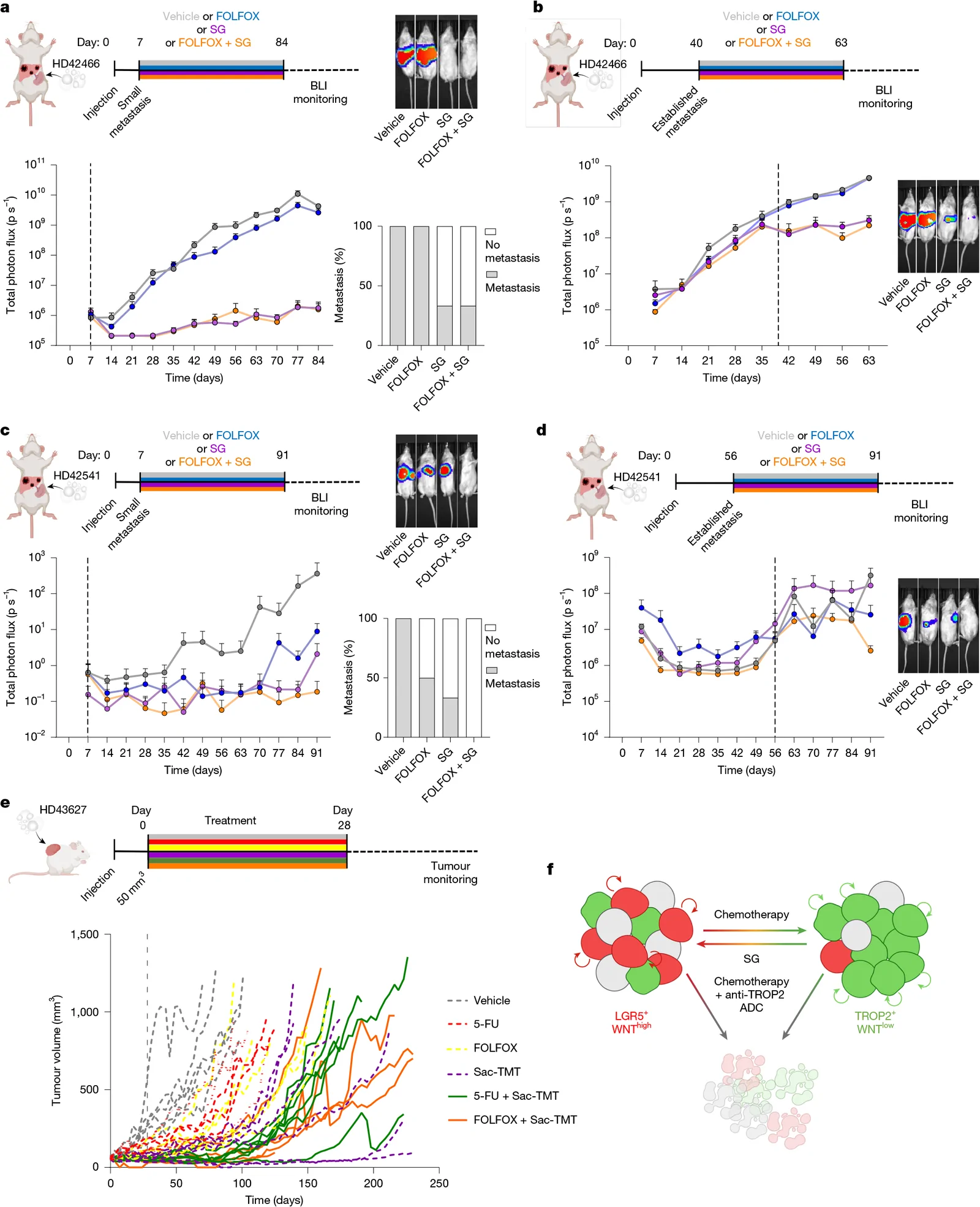
**Combined chemotherapy and TROP2 targeting in CRC.** **a**, Bioluminescence measurements of indicated treatments started at early time points and HD42466 metastasis quantification (*n* = 5, 6, 6 and 6). BLI, bioluminescence imaging. **b**, Bioluminescence measurements of indicated treatments started at late time points and HD42466 metastasis quantification (*n* = 6, 6, 6 and 6). **c**, Bioluminescence measurements of indicated treatments started at early time points and HD42541 metastasis quantification (*n* = 6, 6, 6 and 6). **d**, Bioluminescence measurements of indicated treatments started at late time points and HD42541 metastasis quantification (*n* = 6, 6, 6 and 6). **e**, Schematic indicating the in vivo treatment regimen starting at HD43627 tumour size of 50 m^3^ for 28 days followed by tumour growth monitoring (*n* = 6, 6, 6, 6, 6 and 5). **f**, Summary of the key findings from this study, demonstrating cell-state-dependent CSC properties of CRC and therapy-induced cell state transitioning between WNT^hi^LGR5^+^ and WNT^low^TROP2^+^ cells as a mechanism of resistance. The experimental end point in **a**–**d** was on the last day of treatment as stated in the corresponding scheme. Schematics created in BioRender: **a**–**d**, Jackstadt, R. https://biorender.com/5t4xy1a (2026); **e**, Jackstadt, R. https://biorender.com/3w435x2 (2026); **f**, Jackstadt, R. https://biorender.com/wi6om3c (2026). Source data

**Extended Data Fig. 11:**
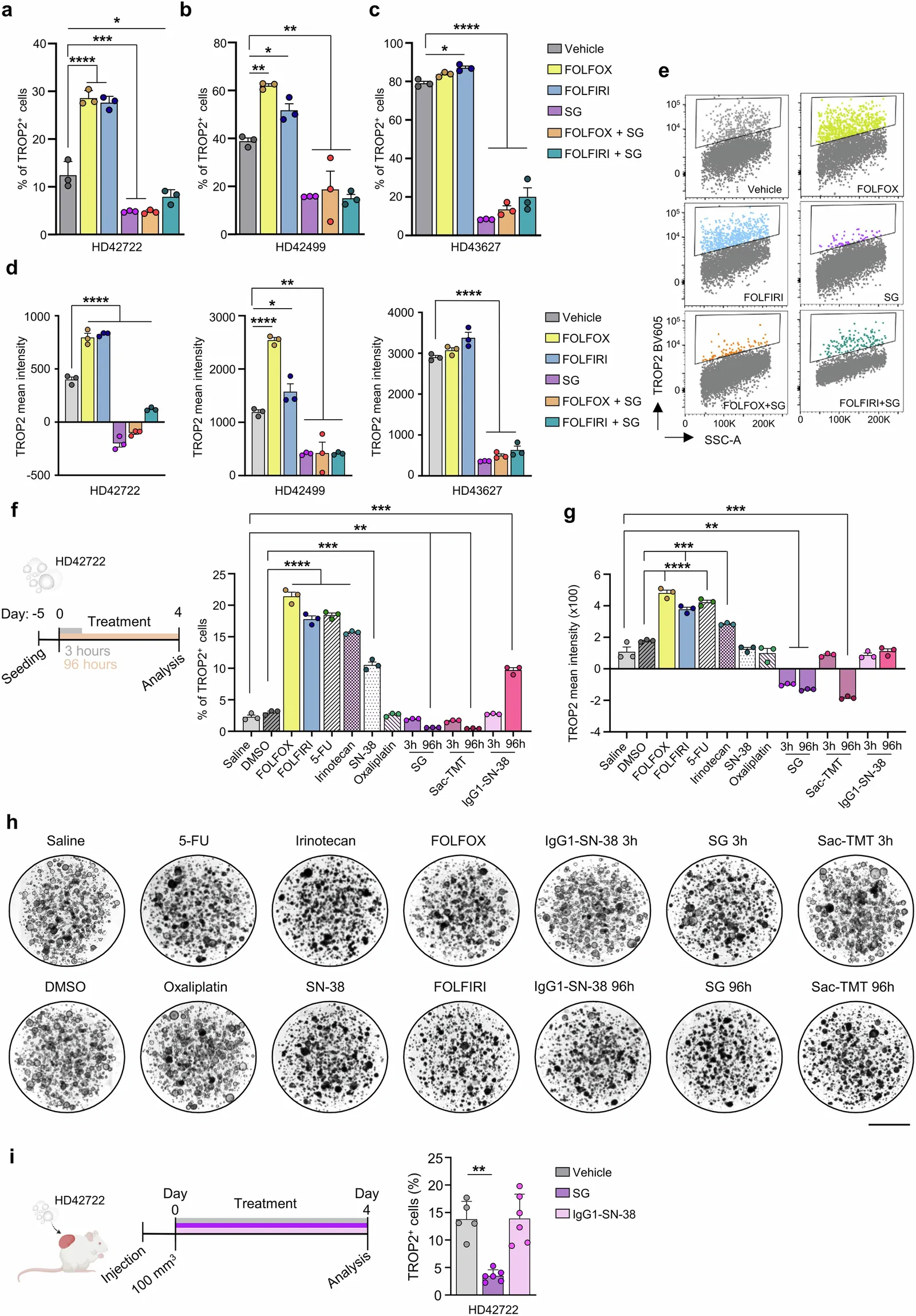
Therapy-mediated regulation of TROP2 expression in colorectal cancer. **a**, **b** and **c**, Quantification of flow cytometry analysis of TROP2^+^ cell abundance 96 h after treatment of PDOs **a** HD42722 (n = 3) **b** HD42499 (n = 3) and **c** HD43627 (n = 3). Representative experiment of at least two independent experiments. **d**, Quantification of TROP2 mean intensity in PDOs as indicated in **a–c**. **e**, Representative flow cytometry dot plots from **a–c**. **f**, Quantification of TROP2^+^ cells upon indicated treatments for 3 or 96 h (n = 3). **g**, Quantification of TROP2 mean intensity upon treatments for 3 or 96 h (n = 3). **h**, Representative pictures of PDOs treated with indicated compounds for 96 h as described in **f**. Scale bar 2000 µm. **i**, Quantification of TROP2^+^ cells after treatment for 4 days. Vehicle n = 5; SG n = 6; IgG-SN-38 (untargeted ADC) n = 6. Statistical analysis in **a-d**, **f**, **g**, **i** was performed using unpaired t-test. Schematics created in BioRender: **f**, Jackstadt, R. https://biorender.com/3w435x2 (2026); **i**, Jackstadt, R. https://biorender.com/3w435x2 (2026).

**Extended Data Fig. 12:**
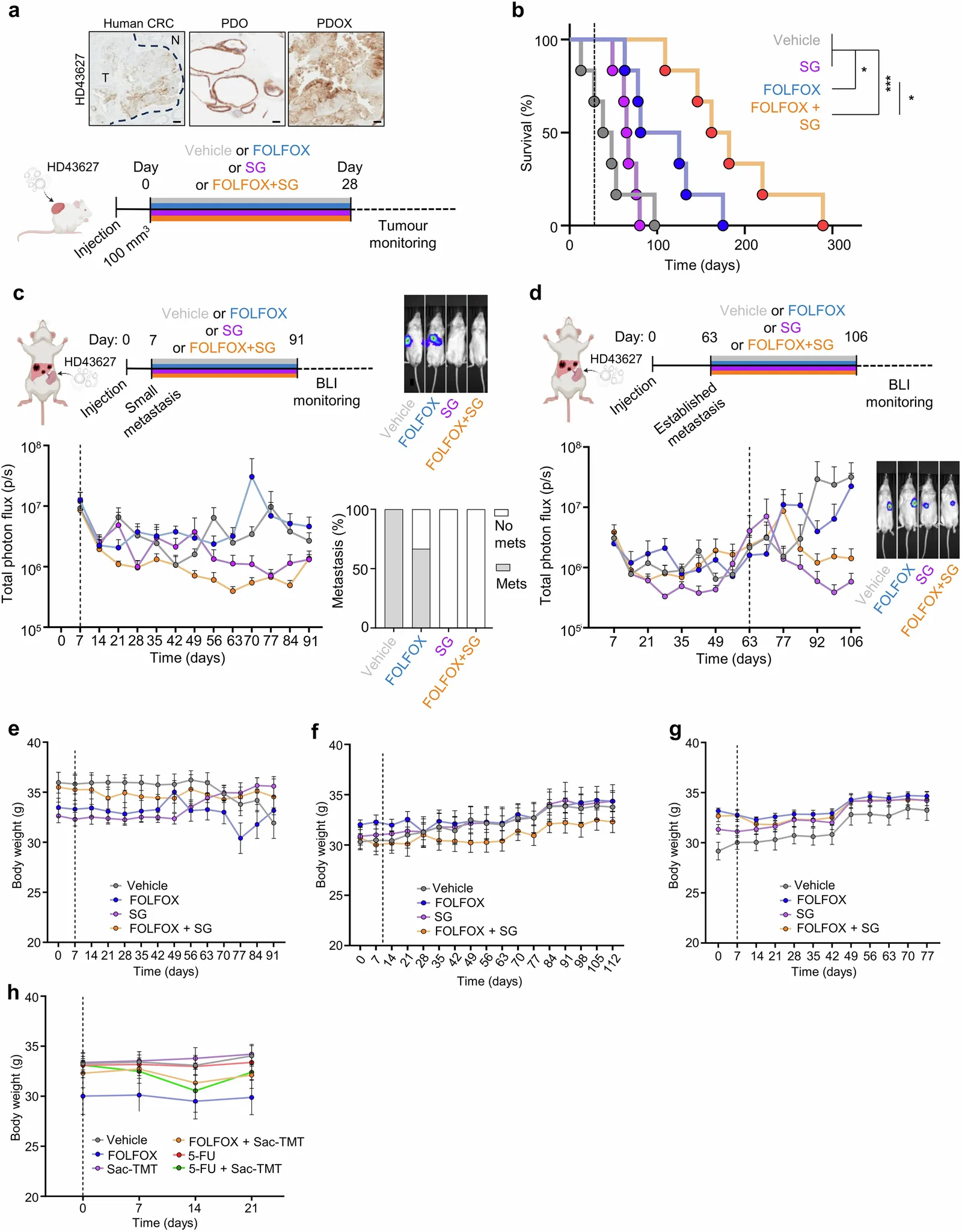
Effects of combined chemotherapy and TROP2-targeting in colorectal cancer. **a**, Top: Immunohistochemistry of TROP2 in patient samples, patient derived organoids (PDOs) and patient derived organoid xenografts (PDOXs). Scale bar 100 µm. Bottom: Cartoon indicating the in vivo treatment regimen. T=tumour, N=normal. **b**, Kaplan-Meier plot of the experiment described in a. Dashed line indicates treatment end. Statistical analysis was performed using the log-rank (Mantel–Cox) test. **c**, Bioluminescence measurements of indicated treatments started at early time points and metastasis quantification (n = 6, 6, 6, 6). **d**, Bioluminescence measurements of indicated treatments started at late time points and metastasis quantification (n = 6, 6, 6, 6). **e**–**h**, Body weights of mice treated with indicated compounds. **e**, in relation to Fig. 5a (n = 5, 6, 6, 6). **f**, in relation to Fig. 5c (n = 6, 6, 6, 6). **g**, in relation to Extended Data Fig. 12c (n = 6, 6, 6, 6). **h**, in relation to Fig. 5e (n = 6, 6, 6, 6, 6, 5). The experimental endpoint in **c-d** was on the last day of treatment as stated in the corresponding scheme. Schematics created in BioRender: **a**, Jackstadt, R. https://biorender.com/3w435x2 (2026); **c**,**d**, Jackstadt, R. https://biorender.com/5t4xy1a (2026). Source data

We demonstrate that chemotherapy induces TROP2 expression, consistent with a therapy-induced cell state associated with resistance. Thus, we propose that combinatorial treatment with chemotherapy and SG might enhance therapeutic efficacy. To test this, we used a range of PDOs that expressed TROP2 at different basal abundance. FOLFIRI or FOLFOX treatment significantly enhanced TROP2 expression (Extended Data Fig. 11a–i). Both SG monotherapy and combination therapy resulted in a significant reduction of surface TROP2-expressing cells (Extended Data Fig. 11a–e). Upon transplantation of PDOs, we detected significantly increased survival of mice treated with FOLFOX and SG in comparison to each monotherapy (Extended Data Fig. 12a,b). Treatment of early metastasis versus established macro-metastasis revealed a TROP2-expression-dependent efficacy with strong effects on the TROP2^hi^ cells (Fig. 5a,b and Extended Data Fig. 12c,d). Combinatorial treatment of either early or established metastasis resulted in reduced bioluminescence signal in TROP2^low^ cells (Fig. 5c,d). Similarly, when we combined monotherapy or combinatorial standard-of-care chemotherapy with Sac-TMT, we observed delayed tumour outgrowth with no toxicity detected (Fig. 5e and Extended Data Fig. 12e–h). These data suggest that combination treatment with these drugs enhances tumour cell killing. In sum, these findings support a model in which LGR5^+^ and TROP2^+^ tumour cell populations co-exist in distinct states. Chemotherapy preferentially targets LGR5^+^ cells and induces TROP2 expression, resulting in a shift in tumour cell state. This creates an opportunity for directed targeting of TROP2^+^ cells. Our data support a therapeutic strategy in which chemotherapy combined with TROP2^+^ cell targeting enhances efficacy through cell-state-specific tumour cell killing (Fig. 5f).

## Discussion

By defining the cellular composition of CRC, we identified the cell surface receptor TROP2 as a marker of poor prognosis. Our data support a scenario in which TROP2 is a combined marker for both fetal and HRC populations, which have been associated with therapy resistance and metastasis. We define a therapeutic strategy to target TROP2-expressing cells using TROP2-directed ADCs. In addition to efficient tumour cell killing, we found that ADCs shape cellular states in the cancer cell continuum, resulting in increased ISC states. Targeting TROP2^+^ cells in combination with standard-of-care chemotherapy shows that TROP2 induction by chemotherapy enhances therapeutic efficacy.

Transcriptional analysis indicates that TROP2 and LGR5 mark distinct cellular populations. Notably, TROP2^+^ cells exhibit CSC properties in WNT^low^ and LGR5^low^ CRCs, supporting the notion that TROP2 marks an alternative CSC pool when LGR5^+^ cells are absent or depleted. In line with previous results in a lineage depletion assay^39^, our data show that LGR5^+^ cells have no bona fide CSC properties in serrated CRC. WNT activation through *Apc *deletion rescued this phenotype. This observation aligns with previously reported increased expression of fetal-like markers in serrated adenoma^40,41^. Tumour induction of *APC*-mutant tubular adenomas occurs through WNT-driven stem cell expansion, whereas serrated adenomas arise from differentiated cells undergoing gastric metaplasia^40^. Whether this dichotomy defines TROP2-expressing cells as the cell of origin of serrated adenoma warrants further investigations. Nonetheless, TROP2 expression as part of the fetal-like wound repair program in the intestine^42,43^ suggests its potential role in inflammation-driven CRC initiation. Further, our functional analysis of metastasis demonstrates that TROP2 labels MICs independently of WNT-signalling conditions, suggesting that cellular states and dynamics are different between MICs and CSCs.

CRC metastasis and therapy resistance have recently been associated with fetal-like/wound-repair progenitor cell states in CRC^24,44,45^. In addition, HRCs were critical for the metastatic relapse of CRC^22^. We found that in human and mouse CRCs, TROP2 marks cells of both of these highly detrimental cellular populations. This distinguishes TROP2 from previously described markers and makes it an excellent target for therapeutic interventions.

Resistance to ADCs can be mediated by reduced or absent target epitope expression^46^. Consistent with this, our data show that the efficacy of TROP2-directed ADCs depends on TROP2 expression levels. In addition, we demonstrate that ADC-mediated cell-state changes within tumours represent a previously unrecognized therapy resistance mechanism. This is relevant for clinical application and demonstrates the importance of companion diagnostics, particularly in CRC. The shift of cellular populations towards the ISC LGR5^+^ state suggests that cell-state-specific targeting is a critical step in overcoming treatment resistance in CRC. We show that the previously recognized equilibrium of cell polarization in CRC^47^ can be exploited to target cellular states along this continuum and that chemotherapy-induced TROP2 expression provides a clinically relevant avenue for future investigation.

## Methods

### Clinical cohorts

All patients in the M0/M1 case–control cohort were diagnosed with colorectal adenocarcinoma at the Institute of Pathology, Faculty of Medicine, LMU Munich, and were subsequently identified through a systematic database search in collaboration with the Munich Cancer Registry. Patients in the M1 group were histologically or radiologically diagnosed with local recurrence or distant metastasis to any site within a 5-year observation period after resection of a colorectal adenocarcinoma. To build the M0 group, a patient who did not progress within the same time frame was pair-matched to each M1 patient according to sex, age, disease stage, tumour grade and primary tumour site. Available patient and tumour characteristics, as well as survival data, were collected. Patients with secondary malignancies were excluded. The availability of sufficient analysable tumour tissue limited patient numbers to 82 per group. Clinicopathological details are listed in Supplementary Table 1. For the UICC stage II cohort, anonymized CRC specimens from patients who underwent surgical resection at LMU Munich between 1994 and 2017 were obtained from the archives of the Institute of Pathology LMU. Follow-up data were recorded prospectively by the Munich Cancer Registry. Specimens were anonymized. Clinicopathological details are listed in Supplementary Table 2. The studies were approved by the Institutional Ethics Committee of the Medical Faculty of the LMU (approval no. 18-105-UE) and carried out according to the Declaration of Helsinki. For both cohorts, TROP2 expression was detected on 4-µm-thick formalin-fixed paraffin-embedded sections using anti-TROP2 antibody (Abcam; ab227691; clone SP295) at a 1:100 dilution on a Ventana BenchMark ULTRA autostainer instrument. Normal human tonsil epithelia were included as positive controls in each staining run.

PDOs and tissues were established from tumours or normal mucosa obtained at the University Hospital Heidelberg (ethical approval nos. S-136/2021 and S-871/2021), in accordance with ethical guidelines and regulations. All patients provided written informed consent. Clinical features and genetic driver mutations (identified by whole-exome sequencing) are provided in Supplementary Table 4.

Kaplan–Meier plots of disease-free survival and relapse-free survival hazard ratios in 1,336 patients with CRC from 16 publicly available datasets were generated using the Kaplan–Meier Plotter tool (https://kmplot.com/analysis/). The automatic best cut-off was used, incorporating data from a previous study^53^. Results shown in Extended Data Fig. 1h,i were generated using SubtypeExploreR (https://subtypeexplorer.qub.ac.uk/).

### Animal experiments

All mouse experiments were approved by the local authorities of Regierungspraesidium Karlsruhe under animal protocols G-148-20, G-27-22, G-159-22 and G-164-22. Tumour growth was monitored regularly, and mice were euthanized when tumours reached the maximum permitted tumour size or when humane end point criteria were met. These limits were defined in accordance with the approved animal protocols and were not exceeded in any of the experiments. The mice were housed at the DKFZ animal facilities in accordance with the local and latest standards with a 12 h dark and light cycle, a constant temperature (20–24 °C) and humidity (45–65%). They were provided with a rodent-specific diet and water ad libitum.

### *Tacstd2*^*CreERT2*^ transgene generation

The TAG stop codon of the *Tacstd2* gene was replaced with a P2A-CreERT2-FRT-NeoR-FRT cassette. This CreER knock-in allele was generated by homologous recombination of a bacterial artificial chromosome clone in embryonic stem cells. Embryonic stem cells were selected with neomycin for 12 days. Insertion of the donor sequence was verified by PCR with the KAPA Long Range PCR Kit (Roche; KK3502) according to the manufacturer’s guidelines. The PCR primers are detailed in Supplementary Table 5. The positive embryonic stem clone was injected into C57BL/6N blastocysts, followed by embryo transfer into RjOrl:SWISS recipient female mice. The neomycin resistance cassette was removed by crossing the founder mouse with the FLPe mouse strain.

### Virus production

Lenti-X 293T cells (Takara Bio; 632180) were cultured in Dulbecco’s Modified Eagle Medium (DMEM) GlutaMAX (Thermo Fisher Scientific; 31966021) with 10% fetal bovine serum(Thermo Fisher Scientific; A5256701) and 1% penicillin–streptomycin (Sigma; P4458-100ML) up to a confluency of 70%. The Lenti-X cells were transfected using polyethyleneimine (PolyScience; 23966-1) with plasmid of interest (Supplementary Table 7), pMD2.G and psPAX2 plasmids in a molar ratio of 1:1:1. Supernatant was changed 12–24 h after transfection. After centrifugation, the supernatant was removed, the lentiviral pellets were resuspended in ice-cold phosphate buffered saline (PBS) and aliquots were stored at −80 °C.

### Organoid transduction and selection

PDOs were dissociated with TrypLE (Gibco; 12605010) and resuspended in 500 µl of complete medium containing 10 µg ml^−1^ of polybrene (Sigma; TR-1003). Subsequently, 50 µl of the virus was added to the organoid suspension, which was spun down at 32 °C for 30 min and then incubated at 37 °C for 1 h. Transduced PDOs were plated in 70% basement membrane extract (BME). Four to five days after transduction, transduced organoids were selected based on the marker present in the plasmid of interest: FACS or blasticidin (10 µg ml^−1^) (Santa Cruz Biotechnology; sc-495389).

### Mouse lines

Male C57BL/6J mice (7–10 weeks old) were purchased from Janvier Labs. NOD scid IL2Rγ null (NSG) mice were provided by the animal facility at DKFZ. *Lgr5*^*EGFP–IRES–CreERT2*^ (RRID:IMSR_JAX:008875), *Rosa26*^*LSL–tdTomato*^ (B6.Cg-Gt(ROSA)26Sortm14(CAG–tdTomato)Hze/J; RRID:IMSR_JAX:007914), *FLPe* (B6.Cg-Tg(ACTFLPe)9205Dym/J; RRID:IMSR_JAX:005703) and the *Tacstd2*^*CreERT2*^ allele was generated as described above. All GEMMs were bred on a C57BL/6J background. *Lgr5*^*eGFP–IRES–CreERT2*^ and *Tacstd2*^*CreERT2*^ alleles were kept in heterozygosity, whereas *Rosa26*^*LSL–tdTomato*^ and the *FLPe* alleles were used in either homozygosity or heterozygosity. The *Tacstd2*^*CreERT2*^ allele was used only in experiments in which the neomycin resistance cassette had been removed.

### Subcutaneous injection and tumour monitoring

Mice were anaesthetized with isoflurane, and single cells in a final volume of 100 µl (50-µl PBS and 50-µl BME) were injected into the left flank of the mice. Tumour growth was monitored three times a week. The experimental end point was defined by tumour size less than 1.5 cm, tumour ulceration, animal distress or end of specific treatment.

To assess the tumour-initiating capacity of tumour cell populations, a defined number of viable single cells from dissociated allografts or organoids were sorted and transplanted subcutaneously into recipient mice. Experiments were ended after 7–9 weeks for PDOs or once tumours reached the end point for MDOs.

Tumour-initiating capacity was assessed by limiting dilution analysis using the web tool ELDA^54^. Active stem cell frequency was estimated based on the dilution series under a single-hit Poisson model using a generalized linear model and compared across conditions using a likelihood-ratio test.

### Intra-splenic injection

Single cells were injected into the spleen of mice in a final volume of 50-µl PBS. An incision was made on the left flank of the mouse to access the spleen. Metamizole (200 mg kg^−1^) was subcutaneously administered before surgery. Lidocaine (10 mg kg^−1^) and bupivacaine (3 mg kg^−1^) were administered subcutaneously close to the incision site before surgery. Metamizole was administered in the drinking water for 3 days after surgery. The experimental end point for each experiment is stated in the figure legends.

### Tissue processing and histological analysis

Human and murine tissues were fixed overnight in 10% formalin at 4 °C. Paraffin blocks were cut into sections with a thickness of 3–5 μm. Sections were deparaffinized by two consecutive washes with xylene for 3 min each, followed by rehydration.

Metastatic burden was evaluated on haematoxylin-and-eosin-stained sections. The area of each individual metastatic nodule was delineated and annotated. Metastatic burden was calculated as the percentage of liver area occupied by metastatic nodules.

### Immunofluorescence

After tissue processing, antigen retrieval was performed in a steamer using boiling target retrieval solution (Dako; S169984) for 30 min. Next, slides were washed for 5 min in Tris-buffered saline with Tween-20 (TBS-T). Samples were blocked in Tris–NaCl blocking buffer (0.1 M Tris–HCl (pH 7.5) and 0.15 M NaCl with 0.5% w/v blocking reagent (PerkinElmer; FP1020)) for 1–2 h at room temperature and incubated with specific primary antibodies (Supplementary Table 7) either overnight at 4 °C or for 1.5 h at room temperature. After incubation, the slides were washed twice with TBS-T and incubated with the specific secondary antibody (Supplementary Table 7) and DAPI (1:1,000) for 30 min at room temperature. Images were acquired using a ZEISS LSM 710 microscope and analysed with Fiji (ImageJ) or QuPath v.0.4.3.

### In situ hybridization

In situ hybridization was performed using the manual RNAscope 2.5 HD Duplex Detection Kit (Advanced Cell Diagnostics; 322500) strictly according to the manufacturer’s instructions. Staining was performed on formalin-fixed paraffin sections (3–5 μm), which were cut and placed in a 60 °C oven for 2 h before staining. To ensure the quality and integrity of RNA in tissues, a positive control probe (PPIB) and a negative control probe (DAPB) were used. The following probes were used: Mm-Lgr5 (312171), Mm-Tacstd2-C2 (471751-C2), Hs-Tacstd2-C2 (405471), Hs-Lgr5 (311021), Mm-PPIB-C1/POLR2A-C2 (321651) and Hs-PPIB-C1/POLR2A-C2 (321641).

### Western blot

Cell pellets from PDOs were washed with ice-cold PBS, lysed in ice-cold radioimmunoprecipitation assay buffer (Cell Signaling Technology; 9806S) supplemented with EDTA-free protease inhibitor cocktail (Roche; 4693132001) and phosphatase inhibitor cocktail (Roche; 4906837001) and finally incubated for 30 min on ice. Protein concentration was determined with the Pierce BCA Protein Assay Kit (Thermo Fisher Scientific; 23225), using BSA (Thermo Fisher Scientific; 23209) as the standard reference. For protein denaturalization, protein lysates were then boiled for 10 min at 70–95 °C with NuPAGE LDS buffer (Invitrogen; NP0007) and NuPAGE Sample Reducing Agent (Invitrogen; NP0009). Denaturalized proteins were loaded in a precast SDS–PAGE gel (Bio-Rad; 5671084). Next, proteins were transferred to a nitrocellulose (Bio-Rad; 1704159) or a polyvinylidene difluoride (Bio-Rad; 1704157) membrane using a semi-dry transfer method with the Trans-Blot Turbo Transfer System (Bio-Rad; 1704150) following the ‘1.5 MM Gel protocol’. Primary and secondary antibodies are listed in Supplementary Table 7. Finally, membranes were developed by chemiluminescence using horseradish peroxidase substrate (Bio-Rad; 1705060 or 1705062) and imaged with the ChemiDoc Imaging System (Bio-Rad).

### Immunohistochemistry

Following tissue processing, antigen retrieval was performed using Target Retrieval Solution (Dako; S1699). Slides were cooled and incubated in 3% hydrogen peroxide (Sigma; 95321). Sections were incubated in normal swine serum (BIOZOL; LIN-ENP9010-10) at room temperature. Primary antibodies (Supplementary Table 7) were applied either overnight at 4 °C or for 1 h at room temperature. After incubation, the slides were rinsed with 0.1% TBS-T and incubated for 30 min to 1 h at room temperature together with the appropriate biotin-conjugated secondary antibody (Supplementary Table 7). The slides were washed with 0.1% TBS-T and incubated with the VECTASTAIN ABC Kit (Vector Laboratories; PK-6100) for 30 min at room temperature. The slides were washed again with 0.1% TBS-T. Signal detection was conducted with DAB Chromogen (Agilent Dako; GV82511-2) following the manufacturer’s instructions.

### Quantitative real-time PCR

RNA from a minimum of 40,000 FACS-sorted cells was isolated using the Arcturus PicoPure RNA Isolation Kit (Applied Biosystems; KIT0204) following the manufacturer’s guidelines. Tissue samples or organoids preserved in RNAlater (Invitrogen; AM7021) were homogenized with the gentleMACS homogenizer with RLT buffer in gentleMACS M Tubes (Miltenyi Biotec; 130-096-335). RNA from cell pellets or homogenized tissue was extracted using QIAGEN RNeasy Kit (74104) according to the manufacturer’s instructions.

Complementary DNA was synthesized by reverse transcription of the isolated RNA using the High-Capacity cDNA Reverse Transcription Kit (Applied Biosystems; 4374966) or the SuperScript VILO Master Mix (Thermo Fisher Scientific; 11755050) according to the manufacturer’s guidelines. Quantitative real-time PCR was performed using the PowerUp SYBR Green Master Mix (Thermo Fisher Scientific; A25778) following the manufacturer’s guidelines. Primer sequences for targeted genes are detailed in Supplementary Table 8.

### Tumour dissociation

For tumour dissociation into single cells, tumour fragments were thawed and enzymatically digested with the Tumour Dissociation Kit (Miltenyi Biotec; 130-095-929) following the manufacturer’s instructions. In brief, 5 ml of DMEM GlutaMAX (Thermo Fisher Scientific; 31966021) was supplemented with 500 µl of the enzyme mix and placed in the gentleMACS C Tube (Miltenyi Biotec; 130-096-334) together with the tumour fragments. The tubes were placed in the gentleMACS Octo Dissociator with Heaters (Miltenyi Biotec; 130-096-427) and dissociated using the default programs 37C_m_TDK_1 (for murine tissue) and 37C_h_TDK_1 (for human tissue). Single cells were stained with different specific antibodies.

### FACS and flow cytometry analysis

Single-cell samples from human or mouse organoids and tumours were generated as previously described. Single cells were stained with fluorescent antibody conjugates (Supplementary Table 7) for 30 min at 4 °C in the dark. Next, samples were washed with 5-ml FACS buffer (PBS with 1% fetal calf serum) and resuspended in 200-µl FACS buffer with DAPI (BioLegend; 422801) or Zombie NIR (BioLegend; 423105), which were used as death exclusion markers. The samples were analysed on a BD LSRFortessa and further analysed on FlowJo v.10.7.1. For FACS, the Fusion Cell Sorter was used.

### In vitro drug treatment

FOLFIRI chemotherapy treatment was performed by treating MDOs or PDOs with 5 µM 5-FU (Selleckchem; S1209) and 5 µM irinotecan (Selleckchem; S2217) for 96 h. FOLFOX chemotherapy treatment was performed by treating PDOs with 5 µM 5-FU and 200 nM oxaliplatin (Selleckchem/BIOZOL; S1224) for 96 h. 5-FU treatment was performed by treating PDOs with 5 µM 5-FU for 96 h. Oxaliplatin treatment was performed by treating PDOs with 200 nM oxaliplatin for 96 h. Irinotecan treatment was performed by treating PDOs with 5 µM irinotecan for 96 h. SN-38 treatment was performed by treating PDOs with 1 µM SN-38 (TargetMol; T1703) for 96 h. SG treatment was performed by treating PDOs with 5 µM SG (MedChemExpress; HY-132254) for 3 h or 96 h. Sac-TMT treatment was performed by treating PDOs with 5 µM Sac-TMT (MedChemExpress; HY-164789) for 3 h or 96 h. IgG1–SN-38 treatment was performed by treating PDOs with 5 µM ADC control human IgG1–SN-38 (MedChemExpress; HY-164668) for 3 h or 96 h. Subsequently, cells were collected to assess the effect of the treatment by flow cytometry or quantitative real-time PCR.

### In vivo drug treatment

FOLFIRI chemotherapy treatment was performed by treating mice with 10 mg kg^−1^ of 5-FU (InvivoGen; sud-5fu-4) in 0.9% NaCl 5 days a week and 45 mg kg^−1^ of irinotecan (Merck; PHR2717) in 0.9% NaCl with 5% DMSO twice a week through intraperitoneal injections. FOLFOX chemotherapy treatment was performed by treating the mice with 10 mg kg^−1^ of 5-FU in 0.9% NaCl 5 days a week and 2 mg kg^−1^ of oxaliplatin (Selleckchem/BIOZOL; S1224) in 0.9% NaCl once a week through intraperitoneal injections. 5-FU treatment was performed by treating the mice with 10 mg kg^−1^ of 5-FU in 0.9% NaCl 5 days a week through intraperitoneal injections. SG (MedChemExpress; HY-132254) treatment was performed by treating the mice with 25 mg kg^−1^ of SG in 0.9% NaCl twice a week through intraperitoneal injections. Sac-TMT (MedChemExpress; HY-164789) treatment was performed by treating the mice with 5 mg kg^−1^ of Sac-TMT in 0.9% NaCl twice a week through intraperitoneal injections. IgG1–SN-38 (MedChemExpress; HY-164668) treatment was performed by treating the mice with 25 mg kg^−1^ of ADC control human IgG1–SN-38 in 0.9% NaCl once through intraperitoneal injection. The starting point and duration of the treatment are indicated for each experiment in the figure legends.

### Lineage tracing

To induce in vitro recombination of the *Rosa26*^*LSL–tdTomato*^ allele, organoids were treated with 10 µM of 4-OHT for 24 h. 4-OHT was removed from the medium, and cells were analysed by flow cytometry to evaluate tdTomato signals at the time points specified in the figure. For lineage tracing of TROP2^+^ cells after chemotherapy treatment, organoids were treated with 10 µM of 4-OHT for 24 h followed by 96 h of FOLFIRI treatment. Cells were analysed by flow cytometry to evaluate tdTomato signals at specified time points. To induce in vivo recombination of the *Rosa26*^*LSL–tdTomato*^ allele, TcT–AKP organoids were dissociated as described, and single cells were used for intra-splenic injections as described above. Mice were treated once with 5 mg kg^−1^ of tamoxifen 2 weeks after MDO injection. Two days after the tamoxifen treatment, the mice were treated with either FOLFIRI (as described before) or vehicle for 2 weeks. To assess the presence of tdTomato clones in the liver metastases of the mice, livers were sampled as indicated in the figure.

### Clonogenicity assays

MDOs or PDOs were dissociated into single cells as previously described. Subsequently, 1,000 MDO cells were seeded in 20-µl BME (R&D Systems; 3433-005-01) in 24-well plates and incubated for 5 days until organoids were formed, and 5,000 PDO cells were seeded in 20-µl BME (R&D Systems; 3433-005-01) in 24-well plates and incubated for 10 days until organoids were formed. The number of organoids per well was quantified under a light microscope, and organoid size was quantified for one representative image per well using the Fiji software. The seeding efficiency of both MDOs and PDOs was calculated by dividing the number of the resulting organoids by the initial number of seeded cells.

### CRISPR–Cas9 knock-in vector generation

A single guide RNA (sgRNA) was designed using the CHOPCHOP web tool (https://chopchop.cbu.uib.no/). SgRNA sequences are detailed in Supplementary Table 9. Cloning of the sgRNA sequence into the pSpCas9(BB)–2A-GFP (PX458) plasmid (Addgene; 48138) was performed as previously described^55^. Insertion of the sgRNA of interest in the backbone was verified by Sanger sequencing (Supplementary Table 9).

To introduce the tdTomato cassette into the *Lgr5* locus, the HR180–LGR5 vector (Addgene; 129094) was used. To that end, the 5′ and 3′ homology arms for the human *LGR5* locus in the HR180–LGR5 original vector were replaced for the homology arm specific for the mouse *Lgr5* locus. The homology arms were amplified from C57BL/6J genomic DNA (gDNA) with primers containing restriction-site-specific overhangs (Supplementary Table 9). The 5′ homology arm and vector were digested with NdeI (Thermo Fisher Scientific; ER0582) and SacI-HF (New England Biolabs; R3156S) (for *Lgr5*) or with NdeI and NsiI-HF for 1 h at 37 °C. The digested backbone was gel-purified, and the digested 5′ homology arm was PCR-purified for a sequential ligation using the T4 ligase as previously described. Next, the 3′ homology arm and vector were digested with AflII and SphI restriction enzymes for 1 h at 37 °C. The digested backbone was gel-purified (QIAGEN; 28706), and the digested 3′ homology arm was PCR-purified (QIAGEN; 28106) for a sequential ligation using the T4 ligase as previously described. The correct insertion of homology arms was verified by Sanger sequencing (Supplementary Table 9).

### CRISPR–Cas9 knock-in generation and selection

VKPN and VAKPS MDOs were electroporated with 5 µg of the plasmid pSpCas9(BB)–2A-GFP (PX458) (Addgene; 48138) and 5 µg of the donor plasmid. After 72 h, electroporated organoids were selected with puromycin (10 µg ml^−1^) (Sigma-Aldrich; P9620) for 4–5 days, and Ruby^+^ clones were isolated with a pipette under a fluorescent microscope and expanded. To validate integration of the donor vector, gDNA was extracted using the DNeasy Blood & Tissue Kit (QIAGEN; 69506) according to the manufacturer’s instructions. The region of interest was PCR-amplified with DreamTaq Green PCR Master Mix (Thermo Fisher Scientific; K1082) following the manufacturer’s instructions. For the 5′-specific integration, a forward primer located upstream of the 5′ homology arm sequence and a reverse primer located in the integrated cassette were used. For the 3′-specific integration, a forward primer located in the integrated cassette and a reverse primer located downstream of the 3′ homology arm sequence were used (primer sequences are detailed in Supplementary Table 9). For the removal of the Ruby–puromycin resistance cassette, the clones were electroporated with the AAV–CAG–GFP–Cre vector^56^. After 72 h, electroporated organoids were dissociated into single cells and isolated for GFP^+^. GFP^+^ cells were plated and incubated for 1 week in complete medium supplemented with 10 µM Y-27632 (Hölzel Biotech; M1817). Once single-cell-derived organoids were formed, isolation was performed with a pipette under a light microscope. To confirm the floxing of the Ruby–puromycin resistance cassette, gDNA was extracted and the locus was PCR-amplified (primer sequences are detailed in Supplementary Table 9).

### CRISPR–Cas9 knockout generation and selection

For the generation of *Apc* or *Trp53* knockout, murine small intestine-derived organoids were electroporated with the pSpCas9(BB)–sgRNA–2A-GFP vector. To select *Apc* mutant KPN^LT^ cells, GFP^+^ cells were sorted 48–72 h after electroporation with the pSpCas9(BB)–sgRNA(*Apc*)–2A-GFP vector. Single clones were isolated and expanded. To select TcT small intestine organoids with mutations in *Apc*, R-spondin (AVSBio; R001-1) was withdrawn from the medium. To select organoids with mutations in *Trp53*, organoids were treated with 5 ng ml^−1^ of nutlin-3 (MedChemExpress; HY-10029) for 1 week. To validate the knockout efficiency, gDNA was extracted. Respective loci with matched sgRNA sequence annealing were PCR-amplified (primer sequences are detailed in Supplementary Table 9). The amplified region was analysed by Sanger sequencing, and the genome editing efficiency was determined with Synthego (https://ice.synthego.com/#/).

### Mouse-derived organoids

MDOs were generated from intestinal tumours of GEMMs. MDOs were cultured in Cultrex reduced growth factor basement membrane (BME; R&D Systems; 3433-005-01) and grown in Advanced DMEM/F-12 (Gibco; 12634028) supplemented with 1% l-glutamine (Thermo Fisher Scientific; 25030024), 1% HEPES (Sigma; h0887-100), B-27 (Gibco; 12585010), N-2 (Gibco; 17502048) and 1% penicillin–streptomycin (Sigma; P4458-100ML). Depending on the mutational background of the MDOs, the basic medium was additionally supplemented with 50 ng ml^−1^ of epidermal growth factor (EGF) (PeproTech; AF-100-15-1000), 100 ng ml^−1^ of Noggin (PeproTech; 250-38-100) or 100 ng ml^−1^ of R-spondin conditioned medium (AVSBio; R001-1) (Supplementary Table 10).

### Small intestine organoids

Organoids derived from the small intestine were generated as previously described^57^. Small intestinal organoids were maintained in basic medium supplemented with 50 ng µl^−1^ of EGF (PeproTech; AF-100-15-1000), 100 ng ml^−1^ of Noggin (PeproTech; 250-38-100) and 1 µg µl^−1^ of recombinant R-spondin conditioned medium (AVSBio; R001-1).

### Patient-derived organoids

CRC and normal colon PDOs were obtained from tumours surgically removed at the University Hospital Heidelberg. Clinical features and genetic driver mutations (identified by whole-exome sequencing) are described in Supplementary Table 4. Tumour-derived cells were grown as organoids in 70% BME and cultured in 50% Advanced DMEM/F-12 (Gibco; 12634028) supplemented with 1% l-glutamine (Thermo Fisher Scientific; 25030024), 1% HEPES (Sigma; h0887-100), 1% penicillin–streptomycin (Sigma; P4458), 50% WRN conditioned medium (generated as previously described^58^), B-27 (Thermo Fisher Scientific; 12587010), N-2 (Thermo Fisher Scientific; 17502048), 1 mM *N*-acetyl-l-cysteine (Sigma; A7250), 10 nM gastrin I (Sigma; G9145), 50 ng µl^−1^ EGF (PeproTech; AF-100-15), 10 µM Y-27632 (Hölzel Biotech; M1817), 0.5 µM A83-01 (Sigma; SML0788), 100 ng µl^−1^ of IGF-1 (BioLegend; 590908), 50 ng ml^−1^ of basic fibroblast growth factor (PeproTech; 100-18b), 4 mM nicotinamide (Sigma; N0636) and 2 µl ml^−1^ Primocin (InvivoGen; ant-pm-2). The medium was changed every other day. PDOs were split once a week using TrypLE (Gibco; 12605010). Normal colon cells were grown as organoids in 70% BME and cultured in 50% Advanced DMEM/F-12 (Gibco; 12634028), supplemented with 1% GlutaMAX Supplement (Thermo Fisher Scientific; 35050061), 1 M HEPES (Thermo Fisher Scientific; 15630080), 1% penicillin–streptomycin (Sigma; #P4458), LRSPO3-Fc fusion protein conditioned medium (AVSBio; R001-1), LNoggin-Fc fusion protein conditioned medium (AVSBio; N002-1), 1× B-27 without vitamin A (Thermo Fisher Scientific; 12587010), 1 mM *N*-acetyl-L-cysteine (Sigma; A9165), 10 mM nicotinamide (Sigma; N0636), 50 ng ml^−1^ of EGF (PeproTech; AF-100-15), 0.5 mM A83-01 (Tocris; 2939), 10 µM SB202190 (Tocris; 1264), 1 µM PGE2 (Tocris; 2296), 0.5 nM WNT Surrogate-Fc Fusion Protein (AVSBio; N001-1) and 2 µl ml^−1^ of Primocin (InvivoGen; ant-pm-2).

### Whole-exome sequencing

Whole-exome sequencing libraries of paired tumour and healthy organoids were generated using the SureSelectXT Automation Reagent Kit and SureSelectXT Human All Exon V7 Capture Library (Agilent Technologies) following the manufacturer’s instructions. In brief, 200 ng of gDNA was fragmented to approximately 150 bp using a Covaris LE220 ultrasonicator. Subsequently, library preparation was performed on a Bravo Automated Liquid Handler (Agilent Technologies), including end-repair, A-tailing, adaptor ligation and amplification. The concentration of amplified, adaptor-ligated DNA library was determined using the TapeStation (Agilent Technologies). In the subsequent steps, 750 ng of amplified, adaptor-ligated DNA library was used for the hybridization reaction with the SureSelectXT All Exon V7 bait set. The DNA library/bait hybrids were captured using streptavidin-coated magnetic beads (Dynabeads MyOne Streptavidin T1; Thermo Fisher Scientific). Index tags were added in the course of PCR amplification of the captured libraries. The final libraries were validated using Qubit (Invitrogen) and TapeStation (Agilent Technologies). Paired-end sequencing (2×100 bp) was performed on an Illumina NovaSeq 6000 according to the manufacturer’s protocol.

The resulting datasets were aligned and called for single-nucleotide variants and indels as described before^59^. In brief, alignment was performed by BWA-MEM. Single-nucleotide variants were called using Samtools, and indels were identified with Platypus using public workflows^60^ (https://github.com/DKFZ-ODCF/IndelCallingWorkflow and https://github.com/DKFZ-ODCF/SNVCallingWorkflow). PDOs were paired against normal tissues from the same donor for somatic mutation calling. Variant allele frequencies were calculated, and variants were classified as somatic, somatic_functional, somatic_functional_ncRNA or germline_functional. Variants in the genes *APC*, *TP53*, *KRAS*, *PIK3CA*, *FBXW7*, *SMAD4*, *TCF7L2*, *BRAF*, *NRAS*, *ARID1A*, *ARID2*, *ERBB3*, *AXIN2*, *SMAD2*, *CTNNB1*, *POLE*, *MSH2*, *PTEN*, *PTPRT*, *ATRX* and *ATM* (in analogy to a previous study^61^), classified as somatic or somatic_functional, were extracted and displayed as the most interesting candidates.

### 10× scRNA-seq

Tumour samples were dissociated into single cells as previously described. Samples were multiplexed using hashing antibodies (TotalSeq-B; BioLegend) according to the manufacturer’s protocol. In brief, single cells were resuspended in Fc receptor blocking solution, and the antibodies were added at the recommended concentration. Patient and murine CRC samples were sorted for live cells, whereas in PDOX samples epithelial cells were extracted. Multiplexed samples were pooled and sequenced following the Chromium Next GEM Single Cell 3′ Reagent Kit 3.1 (dual index) protocol.

### Aligning and demultiplexing single-cell RNA-seq reads

Sequencing reads were mapped to the respective reference genome (human and mouse genome assemblies refdata-gex-GRCh38-2020-A and refdata-gex-mm10-2020-A, respectively) using 10x Genomics Cell Ranger^62^ (v.7.1.0) with standard settings. The Cell Ranger multi pipeline, with an assignment confidence of 0.9, was used to demultiplex the reads into their original samples unless indicated otherwise.

The Cell Ranger count pipeline (v.6.1.0) was used to align data from the following experiments while providing hashtag oligo (HTO) barcodes as a feature library: VAKPS–VPKN and PDO FOLFIRI time course. For those datasets, demultiplexing was performed in R using Seurat’s implementation of HTODemux with default settings on the centred log-ratio normalized HTO counts^63,64^. Cells were classified using HTO_max labels, and singlets that could be assigned to a sample were kept for downstream analysis.

### Preprocessing of data

Sample processing and analysis were performed in Python (v.3.10.9) using the scanpy package^65^ (v.1.9.3) unless indicated otherwise. Quality control was performed at the sample level, filtering cells based on their gene count (lower cut-off between 200 and 750; upper cut-off between the 0.95 and 0.995 percentiles of distribution) and mitochondrial content (lower than 15–30%). Genes were filtered based on their expression abundance across cells (minimum expression in three cells). Putative doublets were identified using scrublet (v.0.2.3) and subsequently removed (cut-off at 0.15–0.2). Count matrices were merged, and highly variable genes were identified in a batch-aware manner using scanpy’s highly_variable_genes function with flavour = seurat, choosing the top 2,000 genes that are highly varying across most samples. The MDO data on VAKPS and VKPN were demultiplexed in R, transferred to Python and subsequently preprocessed at the library level.

For the following datasets, sample preprocessing was performed in an analogous manner in R using Seurat: PDO FOLFIRI time course. Preprocessing was done at the library level. Cells with mitochondrial content greater than 20%, feature counts below 2,000 or total counts below 20,000 were removed from the analysis. Cell cycle inference was performed using Seurat’s built-in function CellCycleScoring and subsequently regressed out during the scaling step ScaleData. Gene set scoring was done for gene sets with 5–200 genes detected in the dataset, keeping genes expressed in at least 30% of all cells and using the function AddmoduleScore_Ucell (Extended Data Fig. 10l).

### Dimension reduction, clustering and visualization

Principal component analysis was performed on scaled normalized counts of highly variable genes, followed by computation of a *k*-nearest-neighbour graph through scanpy’s built-in function with default settings. Cell clusters were defined using the Leiden algorithm (v.0.10.1), and data were visualized after running UMAP. As indicated, missing values were imputed using MAGIC (v.3.0.0; ref. ^66^).

### Data integration

To minimize the influence of batch effects on the analysis, data integration was performed where indicated as described below. The resulting latent space was subsequently used for neighbour detection and clustering as described above.

The following datasets were integrated using harmonypy (v.0.0.9), with the sample ID as batch variable and a maximum of 30 iterations: biobank PDOX data (related to Fig. 1f), biobank tissue data (related to Supplementary Fig. 1) and SG treatment experiment (related to Fig. 4i). On the biobank PDOX data, harmonypy was run twice: first at tissue level for the identification of tumour cells and second on the subset of all epithelial cells.

### Cell type and cell state annotation

Annotations were based on gene set and marker gene expression patterns. For this, differentially expressed genes were calculated for each cluster compared with all others using scanpy’s rank_genes_group function while using a Wilcoxon rank-sum test with Benjamini–Hochberg correction for multiple testing. The cell cycle state was inferred using marker genes for S and G2/M phases as previously described^67^. For datasets containing non-epithelial cells, cell type annotation was additionally guided by label predictions obtained through CellTypist^68^ (v.1.6.3, models (mouse data, Adult_Mouse_Gut.pkl; human data, Adult_Human_Intestine.pkl and Cells_Intestinal_Tract.pkl)). Based on this annotation and the expression pattern of epithelial cell adhesion molecule, the datasets were subsetted to tumour cells for further analysis. Furthermore, highly variable genes were redefined in the epithelial subsets (*N* = 2,000–3,000), followed by dimension reduction, data integration and clustering as described above.

### Composition analysis of cancer subpopulations

Cells were assigned to a certain subpopulation if the respective marker was expressed (counts greater than 0). The relative abundance of subpopulations across all tumour cells was represented as a bar graph.

### Differential expression analysis

Differential expression analysis was performed after generating pseudobulks from the single-cell data, considering the sample origin and the desired contrast conditions for the grouping. Pseudobulks were generated as a sum of counts using Decoupler^69^ (v.1.8.0) while removing genes expressed in less than ten cells or with less than 15 total counts. Contrasting was performed using Wald tests with a significance threshold of 0.05 (Benjamini–Hochberg false discovery rate) in PyDESeq2 (v.0.4.10; ref. ^70^).

### Gene sets, scores and enrichment analysis

Gene set scores were computed at the single-cell level using the scanpy.tl.score_genes function. Conversely, gene set enrichment analysis was performed at the pseudobulk level on the statistics of differential gene expression results using the Decoupler implementation decoupler.get_gsea_df.

Public gene sets registered in the Molecular Signatures Database^71^ were queried through the previous knowledge database OmniPath^72^ (v.1.0.8). In addition, gene sets for the following attributes were taken from the literature as provided in the respective study (Supplementary Table 11): fetal^33^, ISC/LGR5 (ref. ^52^), high relapse (coreHR/epiHR/allHR/tmeHR) and 5-gene LGR5 (ref. ^22^), label retaining cell^50^, crypt proliferation^73^, proliferation signatures^49,74^, Wnt-On and POLR1A^51^, YAP^48^, YAP_22 (ref. ^75^), LGR5Hi MEX3AHi/-Low^19^, squamous and neuroendocrine^24^, revival SCs SSC2c^76^, immature enterocytes^77^ and basal-like pancreatic cancer^78^. The YAP signature^48^ was extracted as a set of genes that responds both to YAP overexpression and inversely to YAP knockout with a minimum average log_2_ fold change of 1.2.

Where necessary, gene homologues of the target species were derived in R (v.4.3.1) as follows: gene symbol to ENTREZ ID mapping was performed using the organism databases org.Mm.eg.db (v.3.17.0) and org.Hs.eg.db (3.17.0), whereas orthologue mapping was executed through Orthology.eg.db (v.3.17.0).

### Treatment response at cluster level

The effect of treatment on cluster composition was assessed as follows: the relative number of cells was calculated for each condition (treatment or time point) and samples across all clusters. Then, for each cluster, the mean across samples was computed. This value was compared across conditions. In the SG treatment data of subcutaneous tumours, clusters that increase (or decrease) in size by 30% were considered responsive and labelled ‘up’ (or ‘down’). For the in vivo SG treatment time course of liver metastasis, the following cut-offs on the log_2_-transformed fold change were selected: |log_2_FC | > log_2_(2) for the 0–48 h dataset and |log_2_FC | > log_2_(1.5) for the 48–120 h dataset. The enrichment of cells from specific time points in clusters was tested using Fisher’s exact test, as implemented in the SciPy package (v.1.11.4; ref. ^79^).

### Trajectory inference

Trajectory inference was performed using Palantir (v.1.4.0; ref. ^80^). To prevent clusters of negligible size or low quality from driving the diffusion map, data subsetting was performed in the following case: SG in vivo treatment time course (to clusters 0–6). The initial state was set within a cluster with significant enrichment at the starting time point based on the outcome of the Fisher’s exact test. The individual cell within this cluster was chosen based on the diffusion map eigenvectors reflecting the temporal resolution as the cell with minimum or maximum value (depending on directionality). Unless indicated otherwise, the terminal states were automatically derived by Palantir. For the following dataset, terminal state selection was guided based on the experimental time points: SG in vivo treatment time course.

### Public data

The transcriptome data and clinical metadata of the TCGA-COAD cohort were loaded through the TCGAbiolinks package^81–83^ (v.2.25.3) in R (v.4.2.0). Gene expression differences across stages were analysed through pairwise comparisons using a Wilcoxon rank-sum test with Holm *P* value correction for multiple testing using the ggpubr::compare_means function (v.0.6.0).

For the comparison of all fetal genes, the fetal gene set^33^, converted to human homologues, served as a starting point. Genes with significant positive correlation between expression value and clinical stage in at least two out of six comparisons were considered putative progression markers.

The epithelial expression data of the SMC and KUL data cohorts^84^ were normalized using the Seurat package (v.5.0.1). Subsequent analyses were performed as described above (related to Extended Data Fig. 1m).

### Statistics

Statistical analysis related to the analysis of the scRNA-seq data was performed as described in Methods using the respective packages. For further statistical analysis, GraphPad Prism software v.9 was used unless indicated otherwise. Limiting dilution analysis was performed using the web tool ELDA (https://bioinf.wehi.edu.au/software/elda/)^54^ and visualized in GraphPad Prism. Statistical tests and number of replicates are specified in the figure legends. Data are presented as mean ± s.e.m. Statistical significance is indicated as follows: *P < 0.05, **P < 0.01, ***P < 0.001 and ****P < 0.0001.

### Reporting summary

Further information on research design is available in the Nature Portfolio Reporting Summary linked to this article.

## Online content

Any methods, additional references, Nature Portfolio reporting summaries, source data, extended data, supplementary information, acknowledgements, peer review information; details of author contributions and competing interests; and statements of data and code availability are available at 10.1038/s41586-026-10705-2.

## Supplementary information

Supplementary Fig. 1 Uncropped western blot images related to Extended Data Fig. 10c,f. Loading controls were run on the same blot.

Reporting Summary

Supplementary Tables Supplementary Tables 1–11.

## Source data

Source Data Fig. 5

Source Data Extended Data Fig. 12

## Data Availability

Raw and processed data objects are available in ArrayExpress/European Nucleotide Archive under the following accession numbers: CRC cohort tissue sequencing (www.ebi.ac.uk/biostudies/ArrayExpress/studies/E-MTAB-16583?query=E-MTAB-16583) and PDOX sequencing (www.ebi.ac.uk/biostudies/ArrayExpress/studies/E-MTAB-16585?query=E-MTAB-16585), SG treatment of subcutaneous tumours (www.ebi.ac.uk/biostudies/ArrayExpress/studies/E-MTAB-16433?query=E-MTAB-16433), time course of SG treatment of liver metastasis (www.ebi.ac.uk/biostudies/ArrayExpress/studies/E-MTAB-16849?query=E-MTAB-16849), time course of SG treatment of PDOs in vitro (www.ebi.ac.uk/biostudies/ArrayExpress/studies/E-MTAB-16843?query=E-MTAB-16843), time course of FOLFIRI treatment of PDOs (www.ebi.ac.uk/biostudies/ArrayExpress/studies/E-MTAB-16836?query=E-MTAB-16836) and MDO-derived tumours VAKPS and VKPN (www.ebi.ac.uk/biostudies/ArrayExpress/studies/E-MTAB-16835?query=E-MTAB-16835). Source data are provided with this paper.

## Code availability

No new software was developed for the analysis presented in this study. Details of the packages and their versions are provided in Methods. A GitHub repository containing the statistical analysis and code used for data visualization is available here: https://github.com/ORCA-HD/TROP2_in_CRC.
