# Supplementary material for: TROP2 targeting reveals therapy-driven cell state dynamics in colorectal cancer
Source: Nature. 2026 Jul 1;656(8129):1023–33. doi: 10.1038/s41586-026-10705-2 (PMC13518220; doi:10.1038/s41586-026-10705-2)
Supplement: Supplementary file 1 [file 41586_2026_10705_MOESM1_ESM.pdf]

nature portfolio

https://doi.org/10.1038/s41586-026-10705-2

Supplementary information

TROP2 targeting reveals therapy-driven cell state dynamics in colorectal cancer

In the format provided by the authors and unedited

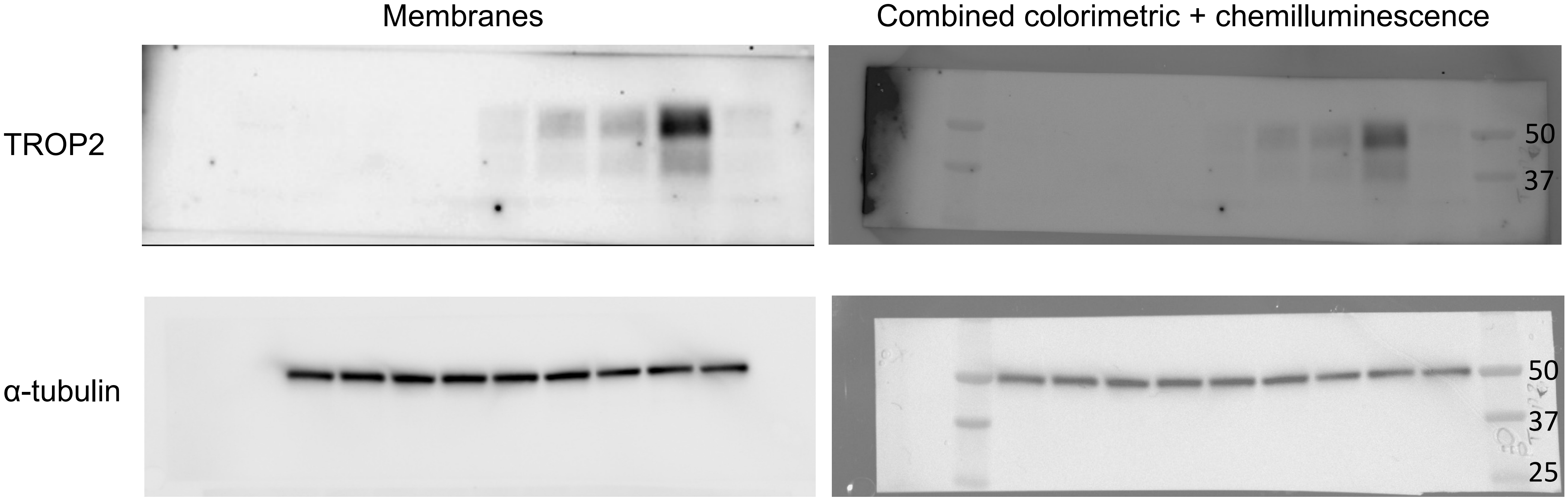

Extended Data Fig. 10c

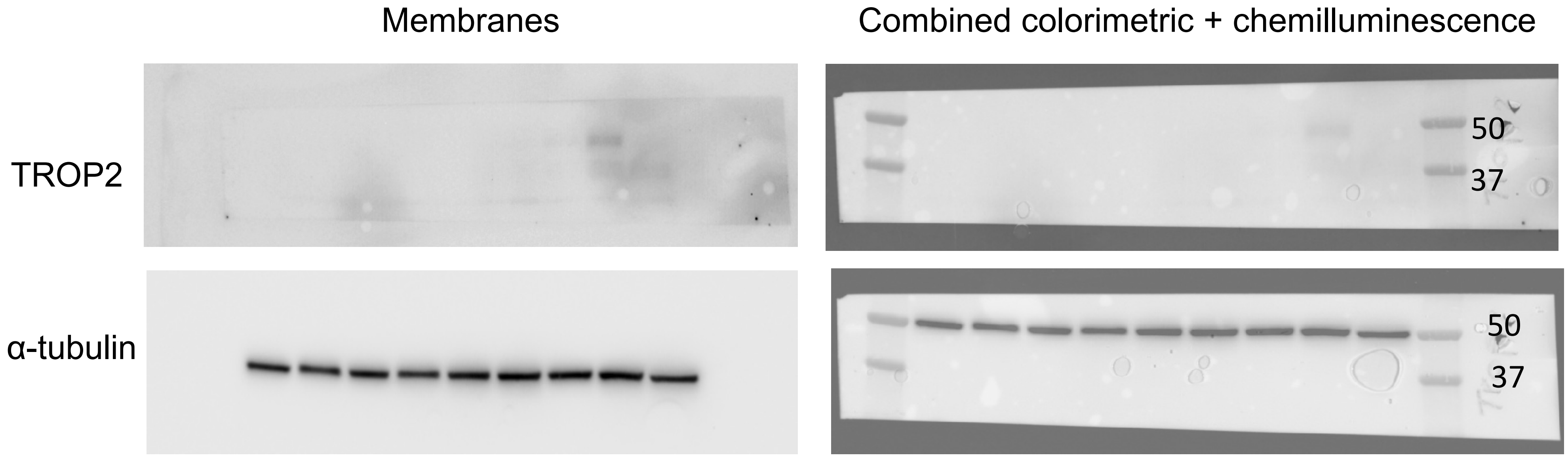

Extended Data Fig. 10f
