# Supplementary material for: TROP2 targeting reveals therapy-driven cell state dynamics in colorectal cancer
Source: Nature. 2026 Jul 1;656(8129):1023–33. doi: 10.1038/s41586-026-10705-2 (PMC13518220; doi:10.1038/s41586-026-10705-2)
Supplement: Supplementary file 2 [file 41586_2026_10705_MOESM2_ESM.pdf]

nature portfolio

Corresponding author(s): Rene Jackstadt

Last updated by author(s): Apr 24, 2026

Reporting Summary

Nature Portfolio wishes to improve the reproducibility of the work that we publish. This form provides structure for consistency and transparency in reporting. For further information on Nature Portfolio policies, see our Editorial Policies and the Editorial Policy Checklist.

Statistics

For all statistical analyses, confirm that the following items are present in the figure legend, table legend, main text, or Methods section.

n/a	Confirmed	
☐	☒	The exact sample size (*n*) for each experimental group/condition, given as a discrete number and unit of measurement
☐	☒	A statement on whether measurements were taken from distinct samples or whether the same sample was measured repeatedly
☐	☒	The statistical test(s) used AND whether they are one- or two-sided<br>*Only common tests should be described solely by name; describe more complex techniques in the Methods section.*
☐	☒	A description of all covariates tested
☐	☒	A description of any assumptions or corrections, such as tests of normality and adjustment for multiple comparisons
☐	☒	A full description of the statistical parameters including central tendency (e.g. means) or other basic estimates (e.g. regression coefficient) AND variation (e.g. standard deviation) or associated estimates of uncertainty (e.g. confidence intervals)
☐	☒	For null hypothesis testing, the test statistic (e.g. *F*, *t*, *r*) with confidence intervals, effect sizes, degrees of freedom and *P* value noted<br>*Give P values as exact values whenever suitable.*
☒	☐	For Bayesian analysis, information on the choice of priors and Markov chain Monte Carlo settings
☒	☐	For hierarchical and complex designs, identification of the appropriate level for tests and full reporting of outcomes
☐	☒	Estimates of effect sizes (e.g. Cohen's *d*, Pearson's *r*), indicating how they were calculated

Our web collection on statistics for biologists contains articles on many of the points above.

Software and code

Policy information about availability of computer code

Data collection

10x single-cell RNA sequencing
Tumor samples were dissociated into single cells as previously described. Samples were multiplexed using hashing antibodies (TotalSeqTM-B, BioLegend) according to manufacturer's protocol. In brief, single cells were resuspended in Fc Receptor Blocking Solution and the antibodies were added at the recommended concentration. Patient and GEMM CRC samples were sorted for live cells whereas PDOX samples were sorted for epithelial cells. Multiplexed samples were pooled and sequenced following the Chromium Next GEM Single Cell 3' Reagent Kit 3.1 (Dual Index) protocol.

Data analysis

Aligning and demultiplexing single-cell RNA-seq reads
Sequencing reads were mapped to the respective reference genome (human and mouse genome assemblies refdata-gex-GRCh38-2020-A and refdata-gex-mm10-2020-A, resp.) using 10X Genomics Cell Ranger44 (v7.1.0) with standard settings. The cellranger multi pipeline with assignment confidence of 0.9 was used to demultiplex the reads into their original samples unless indicated otherwise.
Preprocessing of data
Sample processing and analysis was performed in python (v3.10.9) using the scanpy package45(v1.9.3) unless indicated otherwise. Quality control was performed at sample level, filtering cells based on their gene count (lower cut-off between 200-750, upper cut-off at 0.95 quantile of distribution) and mitochondrial content (lower than 15-30%). Genes were filtered based on their expression abundance across cells (minimum expression in 3 cells). Putative doublets were identified using scrublet (v0.2.3) and subsequently removed (cut-off at 0.15-0.2). Count matrices were merged and highly variable genes were identified in a batch-aware manner using scanpy's highly_variable_genes function with flavour = seurat, choosing the top 2,000 genes that are highly varying across most samples.
Dimension reduction, clustering and visualization
Principal component analysis (PCA) was run on scaled normalized counts of the highly variable genes, followed by computation of a k-nearest-

neighbour graph through scanpy's built-in function with default settings. Cell clusters were defined using the leiden algorithm (v0.10.1) and the data was visualized after running Uniform Manifold Approximation and Projection (UMAP).
Data integration
To reduce the influence of batch effects on the analysis, data integration was performed when indicated as described below. The resulting latent space was subsequently used for neighbor detection and clustering as described above.
The following datasets were integrated using harmonypy (v0.0.9) using the sample ID as batch variable and with a maximum number of 30 iterations: biobank PDOX data (related to Figure 1F), biobank tissue data (related to Figure S1N) and SG treatment experiment (related to Figure 4I). On the biobank PDOX data harmonypy was run twice, first at tissue level for the identification of tumor cells and second, on the subset of all epithelial cells.

For manuscripts utilizing custom algorithms or software that are central to the research but not yet described in published literature, software must be made available to editors and reviewers. We strongly encourage code deposition in a community repository (e.g. GitHub). See the Nature Portfolio guidelines for submitting code & software for further information.

Data

Policy information about availability of data

All manuscripts must include a data availability statement. This statement should provide the following information, where applicable:
- Accession codes, unique identifiers, or web links for publicly available datasets
- A description of any restrictions on data availability
- For clinical datasets or third party data, please ensure that the statement adheres to our policy

Raw and processed data objects have been made available through arrayExpress / European Nucleotide Archive (ENA) under the following accession numbers: CRC cohort tissue-seq (E-MTAB-16583) and PDOX-seq (E-MTAB-16585); SG treatment of subcutaneous tumours (E-MTAB-16433); time course of SG treatment of liver metastasis (E-MTAB-16849); time course of SG treatment of PDOs in vitro (E-MTAB-16843);); time course of FOLFIRI treatment of PDOs (E-MTAB-16836); MDO-derived tumours VAKPS and VKPN (E-MTAB-16835).

Research involving human participants, their data, or biological material

Policy information about studies with human participants or human data. See also policy information about sex, gender (identity/presentation), and sexual orientation and race, ethnicity and racism.

Reporting on sex and gender	Male and female donors were included in this study.
Reporting on race, ethnicity, or other socially relevant groupings	*Please specify the socially constructed or socially relevant categorization variable(s) used in your manuscript and explain why they were used. Please note that such variables should not be used as proxies for other socially constructed/relevant variables (for example, race or ethnicity should not be used as a proxy for socioeconomic status).*<br>*Provide clear definitions of the relevant terms used, how they were provided (by the participants/respondents, the researchers, or third parties), and the method(s) used to classify people into the different categories (e.g. self-report, census or administrative data, social media data, etc.)*<br>*Please provide details about how you controlled for confounding variables in your analyses.*
Population characteristics	Tissue samples from four liver metastases and five primary tumours were used for single cell sequencing and organoid generation. Normal human colon organoids were generated from three individual donors.
Recruitment	*Describe how participants were recruited. Outline any potential self-selection bias or other biases that may be present and how these are likely to impact results.*
Ethics oversight	Tissue collection was approved by the local Ethics committee at Heidelberg University clinics (S-136/2021 and S-871/2021). Patients signed an informed consent.

Note that full information on the approval of the study protocol must also be provided in the manuscript.

Field-specific reporting

Please select the one below that is the best fit for your research. If you are not sure, read the appropriate sections before making your selection.

☒ Life sciences ☐ Behavioural & social sciences ☐ Ecological, evolutionary & environmental sciences

For a reference copy of the document with all sections, see nature.com/documents/nr-reporting-summary-flat.pdf

Life sciences study design

All studies must disclose on these points even when the disclosure is negative.

Sample size	The effect size was determined by prior experimental experience relevant to our study. This estimate informed our power analysis and allowed us to use the sample size that balances statistical power with practical consideration.
Data exclusions	No data points were excluded.

Replication	All in vitro or in vivo experiments were repeated at least twice.
Randomization	Mice were allocated to treatment groups based on tumor size or bioluminescence signal to achieve similar tumour burden between mice.
Blinding	Scientist were not blinded regarding genotype or treatment groups to avoid mislabeling. Experiments were repeated by different independent investigators.

Behavioural & social sciences study design

All studies must disclose on these points even when the disclosure is negative.

Study description	*Briefly describe the study type including whether data are quantitative, qualitative, or mixed-methods (e.g. qualitative cross-sectional, quantitative experimental, mixed-methods case study).*
Research sample	*State the research sample (e.g. Harvard university undergraduates, villagers in rural India) and provide relevant demographic information (e.g. age, sex) and indicate whether the sample is representative. Provide a rationale for the study sample chosen. For studies involving existing datasets, please describe the dataset and source.*
Sampling strategy	*Describe the sampling procedure (e.g. random, snowball, stratified, convenience). Describe the statistical methods that were used to predetermine sample size OR if no sample-size calculation was performed, describe how sample sizes were chosen and provide a rationale for why these sample sizes are sufficient. For qualitative data, please indicate whether data saturation was considered, and what criteria were used to decide that no further sampling was needed.*
Data collection	*Provide details about the data collection procedure, including the instruments or devices used to record the data (e.g. pen and paper, computer, eye tracker, video or audio equipment) whether anyone was present besides the participant(s) and the researcher, and whether the researcher was blind to experimental condition and/or the study hypothesis during data collection.*
Timing	*Indicate the start and stop dates of data collection. If there is a gap between collection periods, state the dates for each sample cohort.*
Data exclusions	*If no data were excluded from the analyses, state so OR if data were excluded, provide the exact number of exclusions and the rationale behind them, indicating whether exclusion criteria were pre-established.*
Non-participation	*State how many participants dropped out/declined participation and the reason(s) given OR provide response rate OR state that no participants dropped out/declined participation.*
Randomization	*If participants were not allocated into experimental groups, state so OR describe how participants were allocated to groups, and if allocation was not random, describe how covariates were controlled.*

Ecological, evolutionary & environmental sciences study design

All studies must disclose on these points even when the disclosure is negative.

Study description	*Briefly describe the study. For quantitative data include treatment factors and interactions, design structure (e.g. factorial, nested, hierarchical), nature and number of experimental units and replicates.*
Research sample	*Describe the research sample (e.g. a group of tagged Passer domesticus, all Stenocereus thurberi within Organ Pipe Cactus National Monument), and provide a rationale for the sample choice. When relevant, describe the organism taxa, source, sex, age range and any manipulations. State what population the sample is meant to represent when applicable. For studies involving existing datasets, describe the data and its source.*
Sampling strategy	*Note the sampling procedure. Describe the statistical methods that were used to predetermine sample size OR if no sample-size calculation was performed, describe how sample sizes were chosen and provide a rationale for why these sample sizes are sufficient.*
Data collection	*Describe the data collection procedure, including who recorded the data and how.*
Timing and spatial scale	*Indicate the start and stop dates of data collection, noting the frequency and periodicity of sampling and providing a rationale for these choices. If there is a gap between collection periods, state the dates for each sample cohort. Specify the spatial scale from which the data are taken*
Data exclusions	*If no data were excluded from the analyses, state so OR if data were excluded, describe the exclusions and the rationale behind them, indicating whether exclusion criteria were pre-established.*
Reproducibility	*Describe the measures taken to verify the reproducibility of experimental findings. For each experiment, note whether any attempts to repeat the experiment failed OR state that all attempts to repeat the experiment were successful.*
Randomization	*Describe how samples/organisms/participants were allocated into groups. If allocation was not random, describe how covariates were controlled. If this is not relevant to your study, explain why.*

Blinding | *Describe the extent of blinding used during data acquisition and analysis. If blinding was not possible, describe why OR explain why blinding was not relevant to your study.*

Did the study involve field work? ☐ Yes ☐ No

Field work, collection and transport

Field conditions | *Describe the study conditions for field work, providing relevant parameters (e.g. temperature, rainfall).*

Location | *State the location of the sampling or experiment, providing relevant parameters (e.g. latitude and longitude, elevation, water depth).*

Access & import/export | *Describe the efforts you have made to access habitats and to collect and import/export your samples in a responsible manner and in compliance with local, national and international laws, noting any permits that were obtained (give the name of the issuing authority, the date of issue, and any identifying information).*

Disturbance | *Describe any disturbance caused by the study and how it was minimized.*

Reporting for specific materials, systems and methods

We require information from authors about some types of materials, experimental systems and methods used in many studies. Here, indicate whether each material, system or method listed is relevant to your study. If you are not sure if a list item applies to your research, read the appropriate section before selecting a response.

Materials & experimental systems

n/a	Involved in the study	
☐	☒	Antibodies
☐	☒	Eukaryotic cell lines
☒	☐	Palaeontology and archaeology
☐	☒	Animals and other organisms
☒	☐	Clinical data
☒	☐	Dual use research of concern
☒	☐	Plants

Methods

n/a	Involved in the study	
☒	☐	ChIP-seq
☐	☒	Flow cytometry
☒	☐	MRI-based neuroimaging

Antibodies

Antibodies used

Anti-human TROP2	Rabbit	Abcam	ab227691
Anti-mouse TROP2	Goat	R&D	AF1122
Anti-RFP	Rabbit	Rockland	600-401-379
Anti-tdTomato	Goat	Sicgen	AB8181-200

List of the biotinylated secondary antibodies used for immunohistochemistry

Antibody	Host Specie	Supplier	Catalog #
anti-rabbit IgG (H+L)	Goat	Vector Laboratories	BA-1000
anti-goat IgG (H+L)	Rabbit	Vector Laboratories	BA-5000

List of the fluorescent secondary antibodies used for IF.

Alexa Fluor 488	anti-rabbit IgG	Goat	Invitrogen	A11034
Alexa Fluor 488	anti-rabbit IgG	Donkey	Invitrogen	A21206
Alexa Fluor 555	anti-goat IgG	Donkey	Invitrogen	A21432
Alexa Fluor 488	anti-rat IgG	Donkey	Invitrogen	A21208

Validation | Validation of antibodies was performed with knock-out cell lines.

Eukaryotic cell lines

Policy information about [cell lines and Sex and Gender in Research](#)

Cell line source(s) | All cell lines were generated in this study.

Authentication | Mouse and human organoids were regularly re-genotyped.

Mycoplasma contamination	Cell lines were tested regularly for mycoplasma contamination with an in-house PCR test. Cells tested negative for mycoplasma contamination.
Commonly misidentified lines (See ICLAC register)	No commonly misidentified cell lines were used in this study.

Palaeontology and Archaeology

Specimen provenance	*Provide provenance information for specimens and describe permits that were obtained for the work (including the name of the issuing authority, the date of issue, and any identifying information). Permits should encompass collection and, where applicable, export.*
Specimen deposition	*Indicate where the specimens have been deposited to permit free access by other researchers.*
Dating methods	*If new dates are provided, describe how they were obtained (e.g. collection, storage, sample pretreatment and measurement), where they were obtained (i.e. lab name), the calibration program and the protocol for quality assurance OR state that no new dates are provided.*

☐ Tick this box to confirm that the raw and calibrated dates are available in the paper or in Supplementary Information.

Ethics oversight	*Identify the organization(s) that approved or provided guidance on the study protocol, OR state that no ethical approval or guidance was required and explain why not.*

Note that full information on the approval of the study protocol must also be provided in the manuscript.

Animals and other research organisms

Policy information about studies involving animals; ARRIVE guidelines recommended for reporting animal research, and Sex and Gender in Research

Laboratory animals	7-10 weeks old male C57BL/6J mice were purchased from Janvier Labs (Saint-Berthevin, France). NOD-scid IL2R gamma null (NSG) mice were provided by the animal facility at DKFZ. Lgr5EGFP-IRES-CreERT2 (RRID:IMSR_JAX:008875); Rosa26LSL-tdTomato (B6.Cg-Gt(ROSA)26Sortm14(CAG-tdTomato)Hze/J; RRID:IMSR_JAX:007914); FLPe (B6.Cg-Tg(ACTFLPe)9205Dym/J; RRID:IMSR_JAX:005703) and the Tacstd2CreERT2 allele was generated as described above. All the GEMMs were bred on a C57BL/6J background. Lgr5eGFP-IRES-CreERT2 and Tacstd2CreERT2 alleles were kept in heterozygosity whereas the Rosa26LSL-tdTomato and the FLPe alleles were utilized in either homozygosity or heterozygosity. The Tacstd2CreERT2 allele was only used in experiments when the neomycin resistance cassette was removed.
Wild animals	This study did not include wild animals.
Reporting on sex	Within each experiment sex-matched animals were used and for transplantation experiments donor and acceptor sex was matched.
Field-collected samples	No field collected samples were used in this study.
Ethics oversight	All mouse experiments were approved and tumour by the local authorities of the Regierungspräsidium Karlsruhe, Baden-Wurttemberg, Germany under the animal protocols permit numbers: G-148-20, G-27-22, G-159-22, G-164-22. Tumour growth was monitored regularly, and mice were euthanized when tumours reached a maximum permitted tumor size or when humane endpoint criteria were met. These limits were defined in accordance with the approved animal protocols and were not exceeded in any of the experiments. [RJ1.1]Mice were housed according to the local and latest standards at the DKFZ animal facilities with a 12 hour dark and light cycle, a constant temperature (20-24°C) and humidity (45-65%) and were provided with a rodent-specific diet and water ad libitum.

Note that full information on the approval of the study protocol must also be provided in the manuscript.

Clinical data

Policy information about clinical studies

All manuscripts should comply with the ICMJE guidelines for publication of clinical research and a completed CONSORT checklist must be included with all submissions.

Clinical trial registration	*Provide the trial registration number from ClinicalTrials.gov or an equivalent agency.*
Study protocol	*Note where the full trial protocol can be accessed OR if not available, explain why.*
Data collection	*Describe the settings and locales of data collection, noting the time periods of recruitment and data collection.*
Outcomes	*Describe how you pre-defined primary and secondary outcome measures and how you assessed these measures.*

Dual use research of concern

Policy information about dual use research of concern

Hazards

Could the accidental, deliberate or reckless misuse of agents or technologies generated in the work, or the application of information presented in the manuscript, pose a threat to:

No	Yes	
☐	☐	Public health
☐	☐	National security
☐	☐	Crops and/or livestock
☐	☐	Ecosystems
☐	☐	Any other significant area

Experiments of concern

Does the work involve any of these experiments of concern:

No	Yes	
☐	☐	Demonstrate how to render a vaccine ineffective
☐	☐	Confer resistance to therapeutically useful antibiotics or antiviral agents
☐	☐	Enhance the virulence of a pathogen or render a nonpathogen virulent
☐	☐	Increase transmissibility of a pathogen
☐	☐	Alter the host range of a pathogen
☐	☐	Enable evasion of diagnostic/detection modalities
☐	☐	Enable the weaponization of a biological agent or toxin
☐	☐	Any other potentially harmful combination of experiments and agents

Plants

Seed stocks

Report on the source of all seed stocks or other plant material used. If applicable, state the seed stock centre and catalogue number. If plant specimens were collected from the field, describe the collection location, date and sampling procedures.

Novel plant genotypes

Describe the methods by which all novel plant genotypes were produced. This includes those generated by transgenic approaches, gene editing, chemical/radiation-based mutagenesis and hybridization. For transgenic lines, describe the transformation method, the number of independent lines analyzed and the generation upon which experiments were performed. For gene-edited lines, describe the editor used, the endogenous sequence targeted for editing, the targeting guide RNA sequence (if applicable) and how the editor was applied.

Authentication

Describe any authentication procedures for each seed stock used or novel genotype generated. Describe any experiments used to assess the effect of a mutation and, where applicable, how potential secondary effects (e.g. second site T-DNA insertions, mosiacism, off-target gene editing) were examined.

ChIP-seq

Data deposition

☐ Confirm that both raw and final processed data have been deposited in a public database such as GEO.

☐ Confirm that you have deposited or provided access to graph files (e.g. BED files) for the called peaks.

Data access links
May remain private before publication.

For "Initial submission" or "Revised version" documents, provide reviewer access links. For your "Final submission" document, provide a link to the deposited data.

Files in database submission

Provide a list of all files available in the database submission.

Genome browser session
(e.g. UCSC)

Provide a link to an anonymized genome browser session for "Initial submission" and "Revised version" documents only, to enable peer review. Write "no longer applicable" for "Final submission" documents.

Methodology

Replicates	*Describe the experimental replicates, specifying number, type and replicate agreement.*
Sequencing depth	*Describe the sequencing depth for each experiment, providing the total number of reads, uniquely mapped reads, length of reads and whether they were paired- or single-end.*
Antibodies	*Describe the antibodies used for the ChIP-seq experiments; as applicable, provide supplier name, catalog number, clone name, and lot number.*
Peak calling parameters	*Specify the command line program and parameters used for read mapping and peak calling, including the ChIP, control and index files used.*
Data quality	*Describe the methods used to ensure data quality in full detail, including how many peaks are at FDR 5% and above 5-fold enrichment.*
Software	*Describe the software used to collect and analyze the ChIP-seq data. For custom code that has been deposited into a community repository, provide accession details.*

Flow Cytometry

Plots

Confirm that:

☒ The axis labels state the marker and fluorochrome used (e.g. CD4-FITC).

☒ The axis scales are clearly visible. Include numbers along axes only for bottom left plot of group (a 'group' is an analysis of identical markers).

☒ All plots are contour plots with outliers or pseudocolor plots.

☒ A numerical value for number of cells or percentage (with statistics) is provided.

Methodology

Sample preparation	For tumor dissociation into single cells, tumor fragments were thawed and fragments were enzymatically digested with the tumor dissociation kit (Miltenyi, #130-095-929) following manufacturer's instructions. In brief, 5 ml of DMEM GlutaMax (Thermo Fisher Scientific, #31966021) was supplemented with 500 µl of the enzyme mix and placed in the gentleMACS C Tube (Miltenyi, #130-096-334) together with the tumor fragments. The tubes were placed in the gentleMACS Octo Dissociator with heaters (Miltenyi, #130-096-427) and dissociated using the default programs 37C_m_TDK_1 (for murine tissue) and 37C_h_TDK_1 (for human tissue). Single cells were stained with different specific antibodies.
Instrument	Samples were analysed on a BD LSR Fortessa. For FACS the Fusion cell sorter was used.
Software	FlowJo 10.7.1.
Cell population abundance	*Describe the abundance of the relevant cell populations within post-sort fractions, providing details on the purity of the samples and how it was determined.*
Gating strategy	For all gating strategies single cells were identified FSC-A/FSC-H. Single cells were stained with anti-TROP2 (human: BV605 anti-TROP2 (Waters Biosciences, #743274); BUV395 anti-TROP2 (Waters Biosciences, #74)3278)); (mouse: APC anti-TROP2 (R&D, #FAB1122A)) (1:100), efl450 anti-CD31 (eBioscience, #48-0311-82) (1:1000), PB anti-CD45 (Biolegend, #103126) (1:1000), FITC anti-EpCAM (human: Miltenyi, #130-113-263) (mouse: Miltenyi, #130-123-674) (1:100) for 30 minutes at 4°C in the dark. Next, samples were washed with 5 ml FACS Buffer (PBS with 1% FCS) and resuspended in 200 µl FACS Buffer with DAPI (Biolegend, #422801) or ZombieNIR (Biolegend, #423105), which were used as death exclusion markers. Accordingly TROP2-high/low or Lgr5-high/low cells were identified.

☒ Tick this box to confirm that a figure exemplifying the gating strategy is provided in the Supplementary Information.

Magnetic resonance imaging

Experimental design

Design type	*Indicate task or resting state; event-related or block design.*
Design specifications	*Specify the number of blocks, trials or experimental units per session and/or subject, and specify the length of each trial or block (if trials are blocked) and interval between trials.*
Behavioral performance measures	*State number and/or type of variables recorded (e.g. correct button press, response time) and what statistics were used to establish that the subjects were performing the task as expected (e.g. mean, range, and/or standard deviation across subjects).*

Acquisition

Imaging type(s) | *Specify: functional, structural, diffusion, perfusion.*

Field strength | *Specify in Tesla*

Sequence & imaging parameters | *Specify the pulse sequence type (gradient echo, spin echo, etc.), imaging type (EPI, spiral, etc.), field of view, matrix size, slice thickness, orientation and TE/TR/flip angle.*

Area of acquisition | *State whether a whole brain scan was used OR define the area of acquisition, describing how the region was determined.*

Diffusion MRI ☐ Used ☐ Not used

Preprocessing

Preprocessing software | *Provide detail on software version and revision number and on specific parameters (model/functions, brain extraction, segmentation, smoothing kernel size, etc.).*

Normalization | *If data were normalized/standardized, describe the approach(es): specify linear or non-linear and define image types used for transformation OR indicate that data were not normalized and explain rationale for lack of normalization.*

Normalization template | *Describe the template used for normalization/transformation, specifying subject space or group standardized space (e.g. original Talairach, MNI305, ICBM152) OR indicate that the data were not normalized.*

Noise and artifact removal | *Describe your procedure(s) for artifact and structured noise removal, specifying motion parameters, tissue signals and physiological signals (heart rate, respiration).*

Volume censoring | *Define your software and/or method and criteria for volume censoring, and state the extent of such censoring.*

Statistical modeling & inference

Model type and settings | *Specify type (mass univariate, multivariate, RSA, predictive, etc.) and describe essential details of the model at the first and second levels (e.g. fixed, random or mixed effects; drift or auto-correlation).*

Effect(s) tested | *Define precise effect in terms of the task or stimulus conditions instead of psychological concepts and indicate whether ANOVA or factorial designs were used.*

Specify type of analysis: ☐ Whole brain ☐ ROI-based ☐ Both

Statistic type for inference | *Specify voxel-wise or cluster-wise and report all relevant parameters for cluster-wise methods.*

(See Eklund et al. 2016)

Correction | *Describe the type of correction and how it is obtained for multiple comparisons (e.g. FWE, FDR, permutation or Monte Carlo).*

Models & analysis

n/a	Involved in the study
☐	☐ Functional and/or effective connectivity
☐	☐ Graph analysis
☐	☐ Multivariate modeling or predictive analysis

Functional and/or effective connectivity | *Report the measures of dependence used and the model details (e.g. Pearson correlation, partial correlation, mutual information).*

Graph analysis | *Report the dependent variable and connectivity measure, specifying weighted graph or binarized graph, subject- or group-level, and the global and/or node summaries used (e.g. clustering coefficient, efficiency, etc.).*

Multivariate modeling and predictive analysis | *Specify independent variables, features extraction and dimension reduction, model, training and evaluation metrics.*
